# Bioactive Compounds, Antioxidant Activities, and HPLC Analysis of Nine Edible Sprouts in Cambodia

**DOI:** 10.3390/molecules28062874

**Published:** 2023-03-22

**Authors:** Visessakseth So, Philip Poul, Sokunvary Oeung, Pich Srey, Kimchhay Mao, Huykhim Ung, Poliny Eng, Mengkhim Heim, Marnick Srun, Chantha Chheng, Sin Chea, Tarapong Srisongkram, Natthida Weerapreeyakul

**Affiliations:** 1 Division of Pharmacognosy, Faculty of Pharmacy, University of Puthisastra, Phnom Penh 120204, Cambodia; 2 Division of Toxicology, Faculty of Pharmacy, University of Puthisastra, Phnom Penh 120204, Cambodia; 3 Division of Basic Pharmaceutical Sciences, Faculty of Pharmacy, University of Puthisastra, Phnom Penh 120204, Cambodia; 4 Division of Pharmacology, Faculty of Pharmacy, University of Puthisastra, Phnom Penh 120204, Cambodia; 5 Department of Technology Research and Development, National Institute of Science, Technology and Innovation, Phnom Penh 120601, Cambodia; 6 Division of Pharmaceutical Technology, Faculty of Pharmacy, University of Puthisastra, Phnom Penh 120204, Cambodia; 7 Faculty of Pharmacy, University of Puthisastra, Phnom Penh 120204, Cambodia; 8 Division of Pharmaceutical Chemistry, Faculty of Pharmaceutical Sciences, Khon Kaen University, Khon Kaen 40002, Thailand; 9 Human High Performance and Health Promotion Research Institute, Khon Kaen University, Khon Kaen 40002, Thailand

**Keywords:** seedling growth, bioactive compounds, antioxidants, HPLC, germination

## Abstract

The non-nutritional health benefits of sprouts are unconfirmed. Thus, nine sprout methanolic extracts were tested for phytoconstituents and antioxidant activity. The TPC, TCC, TFC, TAC, and TALC were measured. ABTS and DPPH radical scavenging and ferric-reducing antioxidant power assays were used to assess the antioxidant activity. HPLC detected gallic acid, vanillin, syringic acid, chlorogenic acid, caffeic acid, and rutin in the extracts. The sprout extracts contained six compounds, with caffeic acid being the most abundant. Gallic acid, syringic acid, chlorogenic acid, caffeic acid, vanillin, and rutin were highest in soybean, black sesame, mustard, sunflower, white radish, and black sesame sprouts, respectively. Sunflower sprouts had the highest level of TCC while soybean sprouts had the highest level of TFC, Taiwanese morning glory had the highest level of TPC, mustard sprouts had the highest level of TALC, and black sesame sprouts had the highest level of TAC. Taiwanese morning glories scavenged the most DPPH and ABTS radicals. Colored and white radish sprouts had similar ferric-reducing antioxidant power. Antioxidation mechanisms varied by compound. Our findings demonstrated that sprouts have biological effects, and their short time for mass production offers an alternative food source for health benefits, and that they are useful for future research development of natural products and dietary supplements.

## 1. Introduction

Various sprouted foods come from different types of seed, such as alfalfa, buckwheat, red cabbage, and broccoli sprouts. Consumption of these sprouts has been increasing and they are considered healthy foods. Sprouts grow from seeds during the germination process that involves various physiological processes. Seeds in the early stage of germination absorb water and comprise hydrolytic enzymes. After activation, these enzymes degrade macromolecules (i.e., proteins, polysaccharides, and lipids) into small molecules that can be easily absorbed into the human body [1]. Secondary metabolic pathways in sprouts are also activated during germination. In addition to their crispness, flavor, and aroma, sprouts contain phytochemicals such as sulforaphane, sulforaphene, isothiocyanates, glucosinolates, enzymes, antioxidants, and vitamins [2]. These secondary metabolites exhibit a wide range of functional activities such as anti-oxidation, anti-inflammatory, anti-cancer, and anti-diabetic activities [3,4].

People have become more interested in eating healthy foods as changes in eating habits have been associated with a reduced risk of diseases, particularly those that are oxidative stress-related. Researchers have, thus, focused on free radicals since these are generated by endogenous (normal cell metabolism, auto-oxidation, electron transport system, enzymatic production by peroxidases) and exogenous sources (pollutants, radiation, cigarette smoking, foods and nutrients, drugs, and xenobiotics). Oxidative stress is caused by an excess of free radicals in the body. Oxidative stress plays a role in chronic and degenerative diseases such as cancer, autoimmune disorders, aging, cataracts, rheumatoid arthritis, cardiovascular disease, and neurodegenerative diseases. The human body has several mechanisms to counteract oxidative stress by producing antioxidants [5]. A balance between free radicals and antioxidants is necessary for proper physiological functions. Since humans are continually exposed to oxidant agents endogenously and exogenously, naturally produced antioxidants in situ cannot neutralize free radicals and overcome oxidative stress. Therefore, antioxidants from external sources through foods and/or supplements are required to maintain this balance. Antioxidants from our diet can aid endogenous antioxidants to relieve some oxidative stress [6,7], which is the subject of our research.

The presence of bioactive substances from plant secondary metabolites in plant-based diets are known to have positive health benefits [8,9,10,11]. Numerous epidemiological studies have demonstrated a link between daily plant-based food consumption, and a decrease in the risk factors for chronic diseases such as cardiovascular disease, diabetes, and obesity [12]. Among the promising vegetables, ready-to-eat sprouts reportedly contain high nutrients and phytochemicals. A previous study demonstrated that alfalfa sprouts contain a high content of saponins and other bioactive compounds with anti-oxidant, anti-diabetic, and anti-viral activity [13]. Buckwheat sprouts, usually eaten with noodles by Asian people, possess anti-oxidant, anti-hypocholesterolemic, and neuroprotective functions [14,15]. Brassica vegetables such as red cabbage and broccoli sprouts also contain anti-microbial, anti-cancer, and anti-obesity properties [16]. The germination of sprouts is a low-cost and efficient technique to activate bioactive chemicals in cereals, vegetables, fruits, flowers, and medicinal plant seeds [17].

Sprouts have gained popularity around the world due to their nutritional value, low cost, convenience, and short production time. Soybean and mung bean sprouts have long been popular and widely consumed in Asia. They are mostly served as fresh salad or porridge, but they are also processed into pickles and cooked. With the demand for functional foods to improve immunity, especially during the COVID-19 pandemic, there is an increase in knowledge about the health benefits of sprouts. A diverse range of sprout germination from a wide range of seeds has been grown and is becoming more popular among health-conscious Cambodians. However, there have been few studies on the bioactive compounds and antioxidant activities of different types of sprouts grown in Cambodia. In this study, nine species of sprouts—sunflower, mustard, black sesame, Taiwanese morning glory, mung bean, soybean, white radish, colored radish, and green pea—were investigated for their total phenolic, anthocyanin, flavonoid, chlorophyll, and alkaloid contents, as well as antioxidant activities (detected by DPPH, ABTS, and FRAP assays). High-performance liquid chromatography (HPLC) analysis was used to identify and quantify some phenolics and flavonoid contents by comparing them to six standard compounds. The discussion of phytochemicals found in sprouts focuses on the health benefits. The information obtained can be used to identify promising sprouts for future research and development of healthy food products and pharmaceuticals.

## 2. Results

### 2.1. Extraction Yield

**Figure 1:**
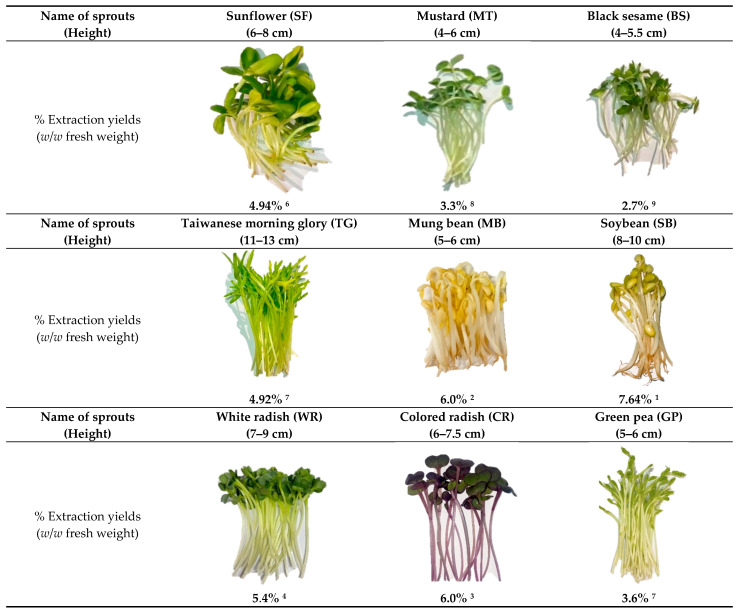
Sprouts at cultivated ages (4–7 days) and different yields. Superscript numbers indicate the rank from high (1) to low (9) % extraction yields.

Different young sprouts were collected in this study, which were *Helianthus annuus* L. (sunflower, SF); *Brassica juncea* (L.) Czern. (mustard, MT); *Sesamum indicum* L. (black sesame, BS); *Ipomoea aquatica* Forssk. (Taiwanese morning glory, TG); *Vigna radiata* (L.) R. Wilczek (mung bean, MB); *Glycine max* (L.) Merr. (soybean, SB); *Raphanus sativus* L. (white radish, WR); *Raphanus sativus* L. (colored radish, CR); and *Pisum sativum* L. (green pea, GP). They were cultivated at the ages of 4–7 days and prepared as methanolic extracts (Figure 1). The rank order from the highest to the lowest percentage of yields is SB (7.64%) > MB (6.01%) > CR (6.0%) > WR (5.4%) > SF (4.94%) > TG (4.92%) > GP (3.6%) > MT (3.32%) > BS (2.68%), respectively (Figure 1).

### 2.2. Identification of Phenolics and Flavonoids Using HPLC

**Figure 2:**
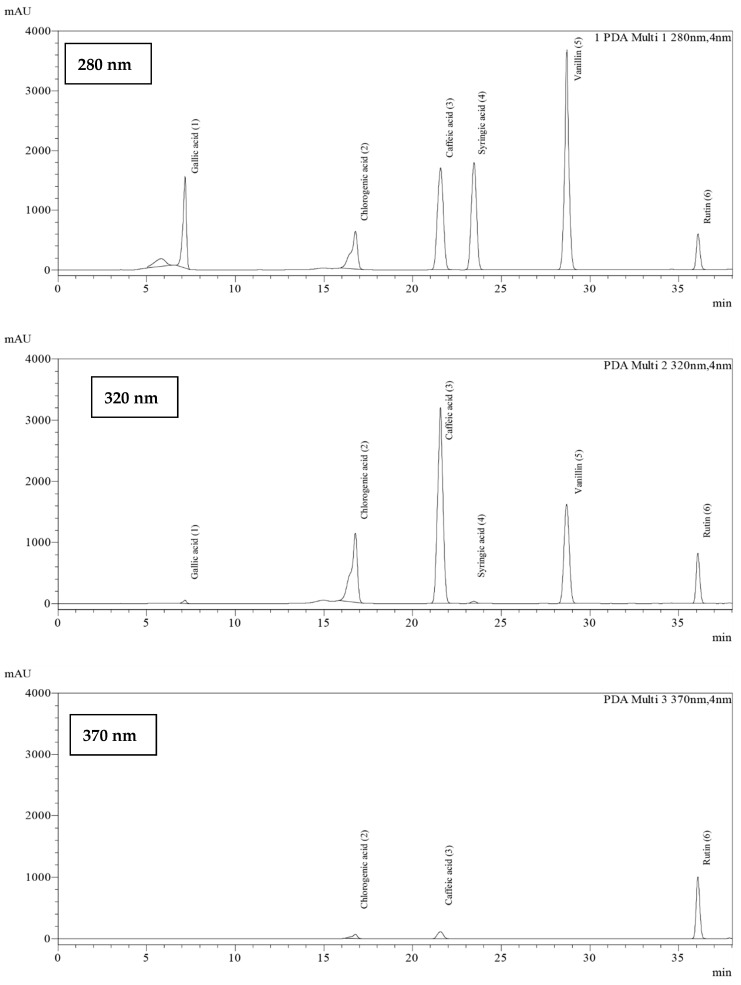
The HPLC chromatogram of the mixture of 6 standard phenolics and flavonoids at a final concentration of 500 µg/mL in methanol was detected at 280, 320, and 370 nm. The peaks are assigned as follows: gallic acid (**1**), chlorogenic acid (**2**), caffeic acid (**3**), syringic acid (**4**), vanillin (**5**), and rutin (**6**).

The phenolic and flavonoid contents in the extracts were identified using HPLC analysis at three different wavelengths (280 nm, 320 nm, and 370 nm) by comparing the retention times with the standards (i.e., gallic acid, syringic acid, chlorogenic acid, caffeic acid, vanillin, and rutin) (Table 1 and Figure 2). The phenolic and flavonoid contents of the sprout extracts were calculated from the peak area compared to those of the standard compounds and the results are shown in Table 2.

Six compounds were identified in the sprout extracts. The highest content of each compound (expressed in milligrams per gram of crude extract) was detected in the plants; gallic acid in SB (4.06 ± 0.01 mg), vanillin in WR (2.03 ± 0.01 mg), chlorogenic acid in MT (2.39 ± 0.01 mg), syringic acid in BS (0.46 ± 0.003 mg), caffeic acid in SF (14.91 ± 0.08 mg), and rutin in BS (5.29 ± 0.02 mg). The phenolic compound with hydroxycinnamic acid structure (i.e., caffeic acid) was the highest amount among all compounds tested and existed in the highest amount in SF. The other hydroxycinnamic acid, chlorogenic acid, was detected as the highest compound compared to other compounds in three sprouts (MT, GP, and CR), while gallic acid was detected as the highest compound in two sprouts (SB and GP). Syringic acid was detected in a small amount in most sprouts except SF and MT, where it was not detected.

### 2.3. Phytochemical Contents

#### 2.3.1. Total Chlorophyll Content (TCC)

**Figure 3:**
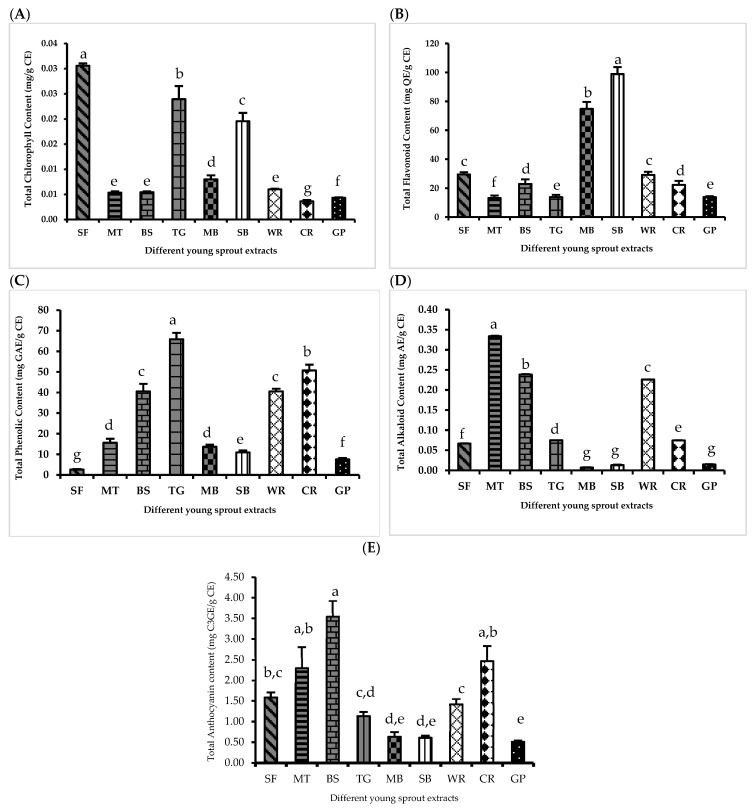
(**A**) TCC, (**B**) TFC, (**C**) TPC, (**D**) TALC, and (**E**) TAC. Data are expressed as mean ± SD from five replicates. Different letters (a–g) indicates a significant difference between samples (*p* < 0.05).

Chlorophyll a and chlorophyll b were detected at 645 and 663 nm, respectively (Figure 3A). The results of the TCC were expressed as milligrams per grams of crude extract. The TCCs in MT, BS, and WR were not significantly different, while the TCCs in the other sprouts were significantly different (*p* < 0.05). SF contained the highest amount of TCC (0.031 ± 0.00045), followed by TG (0.024 ± 0.0026), SB (0.020 ± 0.0016), MB (0.008 ± 0.001), WR (0.006 ± 0.0001), BS (0.006 ± 0.0004), MT (0.006 ± 0.001), GP (0.004 ± 0.000), and CR (0.004 ± 0.000), respectively.

#### 2.3.2. Total Flavonoid Content (TFC)

The total flavonoid content was reported as milligrams of quercetin equivalent (QE) per grams of crude extract (Figure 3B). The sprouts that contained non-significantly different TFCs included a group of WR and SF, BS and CR, and TG and GP. The high to low ranking of flavonoid content was first SB (98.9 ± 4.8), followed by MB (71.8 ± 7.97), WR (29.9 ± 2.43), SF (29.3 ± 1.34), BS (23.6 ± 2.49), CR (21.8 ± 2.61), TG (14.1 ± 1.59), GP (13.6 ± 0.528), and MT (12.5 ± 1.14).

#### 2.3.3. Total Phenolic Content (TPC)

The total phenolic content of various methanolic sprout extracts was determined using Folin-Ciocalteu’s reagents. The results are expressed as milligrams of gallic acid equivalent (GAE) per gram of crude extract (Figure 3C). The sprouts that contained non-significantly different TPCs included a group of BS and WR; and MT and MB. The ranking of the phenolic contents from high to low were TG (65.0 ± 2.60), CR (50.2 ± 2.74), WR (40.6 ± 1.13), BS (40.5 ± 3.67), MT (15.5 ± 1.94), MB (13.6 ± 0.98), SB (11.3 ± 1.06), GP (7.4 ± 0.69), and SF (2.6 ± 0.21), respectively.

#### 2.3.4. Total Alkaloid Content (TALC)

The results of the total alkaloid content were expressed as milligrams of atropine equivalent (AE) per gram of crude extract (Figure 3D). The high to low ranking of alkaloid content was first MT (0.33 ± 0.00), then BS (0.24 ± 0.00), WR (0.23 ± 0.00), TG (0.08 ± 0.00), CR (0.07 ± 0.00), SF (0.07 ± 0.00), GP (0.02 ± 0.00), SB (0.01 ± 0.00), and MB (0.01 ± 0.00). The respective total alkaloid contents of GP, SB and MB were not significantly different.

#### 2.3.5. Total Anthocyanin Content (TAC)

The results of the TAC were expressed as milligrams of cyanidin 3-glycocide equivalent per gram of crude extract (Figure 3E). The high to low ranking of the anthocyanin content was first expressed in BS (3.5 ± 0.38), then CR (2.5 ± 0.37), MT (2.3 ± 0.53), SF (1.6 ± 0.12), WR (1.4 ± 0.13), TG (1.1 ± 0.10), MB (0.6 ± 0.1), SB (0.6 ± 0.1), and GP (0.5 ± 0.03). BS showed a significantly higher TAC than the other sprouts (*p* < 0.05). Furthermore, MB, SB, and GP contained significantly lower TACs than the other sprouts (*p* < 0.05).

### 2.4. Antioxidant Activities

#### 2.4.1. DPPH Radical Scavenging Activities Assay

The results are expressed as the inhibitory concentration at 50% (IC_50_) (Table 3). The low IC_50_ value indicates a high scavenging activity of the extract. Trolox exhibited a DPPH radical scavenging effect with an IC_50_ value of 32.6 ± 4.14 µg/mL. TG exhibited the highest DPPH radical scavenging effect with an IC_50_ value of 283.6 ± 25.87 µg/mL, following by SB (403.5 ± 36.78 µg/mL) (Table 3). The sprouts that possessed non-significantly different DPPH radical scavenging activity included a group of BS (486.3 ± 58.03 µg/mL) and CR (422.70 ± 43.05 µg/mL); and of MB (556.0 ± 18.30 µg/mL) and WR (527.91 ± 14.87 µg/mL). SF and MT exerted significantly weak DPPH radical scavenging activities (IC_50_ values of 1201.01 ± 38.47 and 1480.7 ± 154.93 µg/mL, respectively) (*p* < 0.05).

#### 2.4.2. ABTS Radical Scavenging Activities Assay

**Figure 4:**
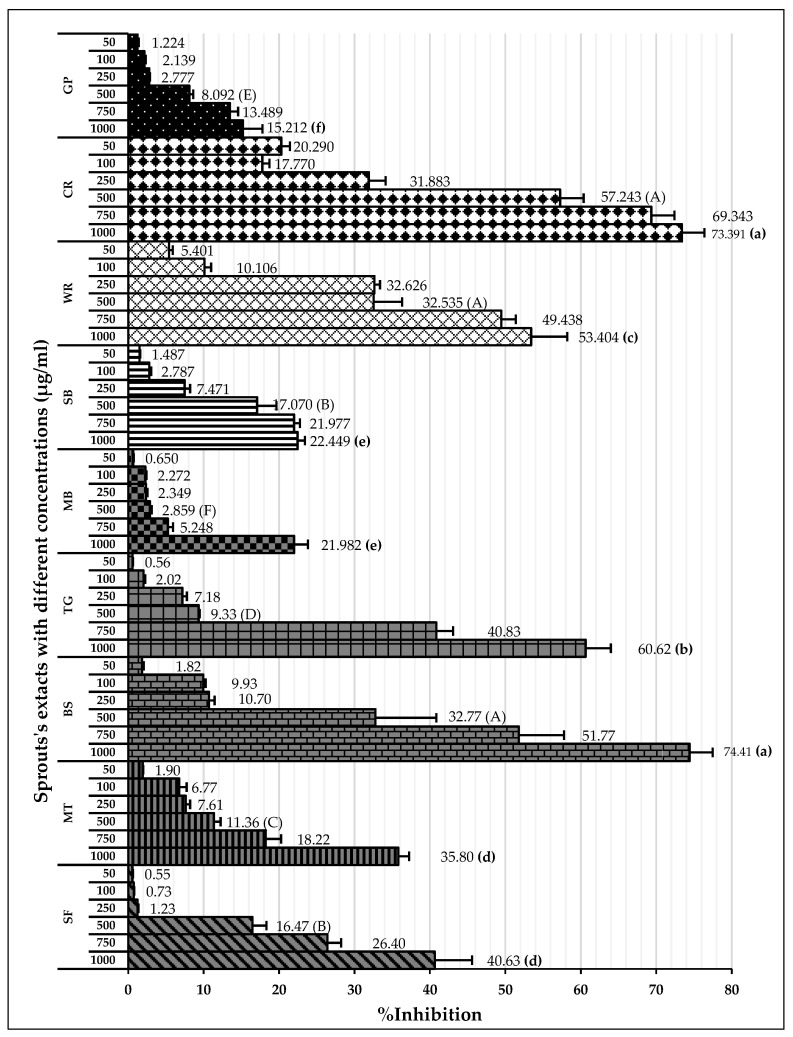
The percent of inhibition of ABTS radicals with different sprouts’ extract. Data are expressed as mean ± SD from five replicates. Different letters (a–f) and (A–F) indicate a significant difference between samples at the same concentration (*p* < 0.05).

The results are expressed as the percent of inhibition. The concentration of the extract used in this study was within the range of 50–1000 µg/mL. The activities were based on concentration (Figure 4). The highest to lowest ABTS radical scavenging activities at 1000 µg/mL were BS (74.4 ± 3.1%), CR (73.4 ± 3.0%), TG (60.6 ± 3.4%), WR (53.4 ± 4.8%), SF (40.6 ± 4.9%), MT (35.8 ± 1.4%), SB (22.4 ± 1.0%), MB (22.0 ± 1.8%), and GP (16.3 ± 1.2%). The inhibitions of the ABTS radical are expressed as IC_50_ values (Table 3). Trolox exhibited an ABTS radical scavenging effect with an IC_50_ value of 44.0 ± 0.680 µg/mL. Among the sprout extracts, TG exhibited the highest ABTS radical scavenging effect with the lowest IC_50_ value.

#### 2.4.3. Ferric-Reducing Antioxidant Power Assay

The single electron transfer mechanism (SET) was indicated by the ferric-reducing antioxidant power assay (FRAP), and this test was performed at a sprout concentration of 500 µg/mL. The result was expressed as the millimoles of FeSO_4_ per gram of dry crude extract. Quercetin, a positive control, showed the highest ferric-reducing antioxidant power (0.56 ± 0.019 mM FeSO4 g^−1^ extract). Among the sprout extracts, CR and WR exerted the highest reducing antioxidant power with non-significantly differences showing 0.059 ± 0.002 and 0.064 ± 0.004 mM FeSO4 g^−1^ extract, respectively, while SB possessed the lowest reducing antioxidant power (0.0044 ± 0.00014 mM FeSO_4_ g^−1^ extract) (Table 3).

### 2.5. The Principal Component Analysis (PCA) of Bioactive Compounds and Antioxidant Activities

**Figure 5:**
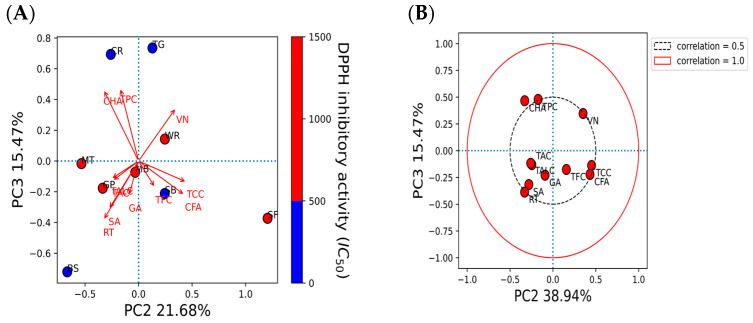

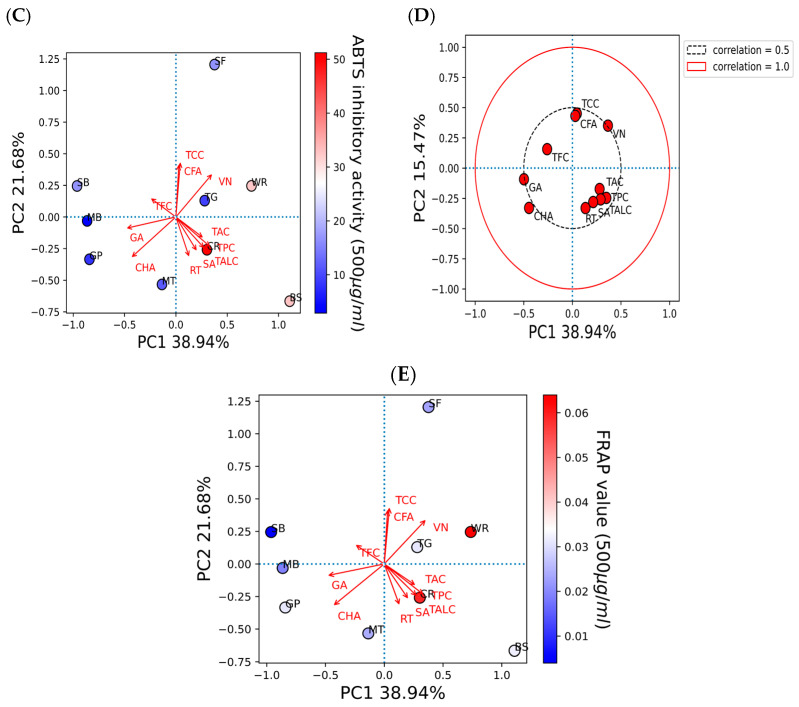
Principal component analysis (PCA) of bioactive compounds in sprout extracts and their antioxidant activities (**A**,**C**,**E**). Score plots between the antioxidant capacity based on DPPH, ABTS, and FRAP assays of SF, MT, BS, TG, MB, SB, WR, CR, and GP (**B**,**D**). Loading plots of bioactive compounds detected in the extracts. Red dot indicates the bioactive compounds. Dashed and solid line indicates correlation >0.5 and 1, respectively. Blue dot in (**A**) indicates the strongest DPPH inhibitory activities. Red dots in (**C**,**E**) indicate the strongest ABTS inhibitory activities and ferric-reducing antioxidant power, respectively. Abbreviation: TCC = total chlorophyll content, TPC = total phenolic content, TAC = total anthocyanin content, TALC = total alkaloid content, TFC = total flavonoid content, GA = gallic acid, SA = syringic acid, CHA = chlorogenic acid, CFA = caffeic acid, VN = vanillin, RT = rutin, DPPH = DPPH radical scavenging activity, FRAP = ferric-reducing antioxidant power, ABTS = ABTS radical scavenging activity.

The correlations of bioactive compounds and the antioxidant capacity of all sprout extracts are presented in Figure 5. Figure 5A,C,E presents the principal analysis between the sprout extracts and their antioxidant activity based on the IC_50_ values inhibiting DPPH radicals, % scavenging of ABTS at 500 µg/mL extract, and the FRAP value at 500 µg/mL extract, respectively. The compounds that contributed to the antioxidant activity are shown using loading plots (Figure 5B,D); namely, TPC, TFC, TCC, TALC, TAC, gallic acid (GA), syringic acid (SA), chlorogenic acid (CHA), caffeic acid (CFA), vanillin (VN), and rutin (RT).

The results in the upper quadrant of Figure 5A showed that TG and CR, which had high DPPH scavenging activities, were separated from the other sprouts above PC3, which explained 15.5% of the variance (Figure 5A). Chlorogenic acid and TPC contributed to the high DPPH scavenging activity of TG and CR based on moderate loading, as shown in Figure 5B. A moderate loading of vanillin indicated that vanillin was the predominant compound found in WR, which had a low activity and separated WR from the other extracts.

BS was in the lower left quadrant, while SB and SF were in the lower right quadrant (explaining 21.7% of the variance) (Figure 5A). Along with PC2, rutin had a moderate loading with BS, which showed a high DPPH scavenging activity (Figure 5B). A higher rutin content was present in BS, with a higher DPPH scavenging activity. The TCC and caffeic acid moderately contributed to the low DPPH scavenging activity of SF.

Figure 5C,D showed a group of sprout extracts (i.e., SB, MB, GP, and MT) with low ABTS inhibitory activity separated from the other groups along PC1 (explaining 38.9% of the variance). Gallic acid and chlorogenic acid contents had a moderate loading with SB, MB, GP, and MT, a group of sprouts with a low ABTS inhibitory activity. These data conformed to the results obtained from the HPLC analysis that gallic acid was high in SB and MB, while chlorogenic acid was high in MT.

The same pattern was observed for the ferric-reducing antioxidant activity of the SB, MB, GP, and MT extracts and the attributed compounds (i.e., gallic acid and chlorogenic acid) by PC1 (explaining 38.9% of the variance) (Figure 5E,D). High gallic acid and chlorogenic acid contents were detected in SB, MB, GP, and MT, a group of sprouts with low ferric-reducing antioxidant activity.

Vanillin was a compound presented in an extract with a high ABTS inhibitory activity and ferric-reducing antioxidant activity of WR along PC1 (Figure 5C–E). Vanillin was the marker found in WR, which exhibited a high ABTS inhibitory activity and ferric-reducing antioxidant activities.

Data suggested that TPC and chlorogenic acid moderately contributed to the stronger DPPH scavenging activity. Vanillin moderately contributed to a moderate to high ABTS inhibitory activity and ferric-reducing antioxidant. Notably, other phytochemicals were not able to discriminate the antioxidants of sprout extracts due to their weak loading or contributions.

### 2.6. Correlation Coefficient (r) of Sprouts at Different Species with Antioxidant Capacity

**Figure 6:**
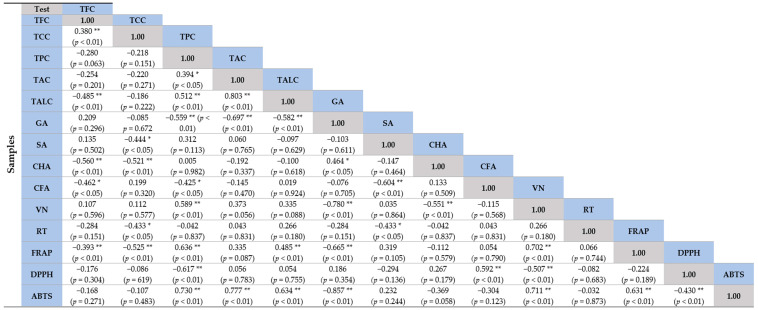
Correlation coefficient (r) between the phytochemicals (TCC, TPC, TFC, TALC, TAC, GA, SA, CHA, CFA, VN, and RT) in different sprouts with antioxidant capacity (DPPH, ABTS, and FRAP). If the r value is close to +1 or −1, it indicates a strong positive relationship or an inverse relationship, respectively. If r is near 0, it indicates a weak or no relationship. * Correlation is significant at *p* < 0.05. ** Correlation is significant at *p* < 0.01. Abbreviation: TCC = total chlorophyll content, TPC = total phenolic content, TAC = total anthocyanin content, TALC = total alkaloid content, TFC = total flavonoid content, GA = gallic acid, SA = syringic acid, CHA = chlorogenic acid, CFA = caffeic acid, VN = vanillin, RT = rutin, DPPH = DPPH radical scavenging activity, FRAP = ferric-reducing antioxidant power, ABTS = ABTS radical scavenging activity.

The correlational characteristics of the nine sprouts are illustrated in Figure 6. We focused on the antioxidant capacities and determined their correlation with bioactive compounds from the chemical screening (i.e., TCC, TPC, TFC, TALC, and TAC) and the HPLC analysis (i.e., GA, SA, CHA, CFA, VN, and RT). A high DPPH scavenging activity was based on a low IC_50_ value.

The results illustrate a significant moderate negative correlation of IC_50_ value from DPPH inhibitory activity with TPC (r = −0.617, *p* < 0.01) and vanillin (r = −0.507, *p* < 0.01). This indicates that TPC and vanillin were present in the extracts with a high DPPH scavenging activity, while vanillin had a high DPPH scavenging activity (low IC_50_ value).

The ABTS scavenging activity, determined from % ABTS inhibition, showed a significant strong positive correlation with the TPC (r = 0.730, *p* < 0.01), TAC (r = 0.777, *p* < 0.01), and vanillin (r = 0.711, *p* < 0.01), respectively. However, this activity had a significantly strong negative correlation with gallic acid (r = −0.857, *p* < 0.01). This indicates that the TPC, TAC, and vanillin existed in the extract with a high % inhibition of ABTS, while gallic acid was present in the extract with a low % ABTS inhibition.

The ferric-reducing antioxidant power was determined from the FRAP value. The higher the value, the better the activity. TPC and vanillin showed a significantly strong positive correlation with the FRAP value (r = 0.636, and 0.702, respectively; *p* < 0.01). Gallic acid, however, had a significantly strong negative correlation with the FRAP value (r = −0.665, *p* < 0.01). TPC and vanillin could be biomarkers in extracts with high reducing powers, while gallic acid could be for low reducing power. This information is in agreement with the data from PCA. Interestingly, rutin does not correlate with any antioxidant capacity of sprout extracts (Figure 6).

Rutin, which is a flavonoid compound, has a weak negative correlation with the TFC. SB comprised the highest TFC but detected gallic acid as the highest compound compared to rutin. This indicates that other flavonoids are also present in SB. All other phenolic compounds identified by HPLC analysis also showed moderate to weak, positive, and negative correlations with the TCC. Data suggest that some unidentified compounds might be present in the sprout extracts.

## 3. Discussion

Reactive oxygen species are formed from endogenous sources (normal cell metabolism, electron transport system, and enzymatic production by peroxidases) or exogenous sources (pollutants, radiation, smoking, nutrients, drugs, and xenobiotics). The overproduction of free radicals in the body likely results in oxidative stress. Oxidative stress plays a big role in chronic and degenerative diseases such as cancer, inflammation, rheumatoid arthritis, cardiovascular, neurodegenerative disease, and aging. Many studies have reported the antioxidant activities of plant extracts. The antioxidant mechanism proceeds through two processes, including: (1) giving the free radical one electron, which is a chain-breaking mechanism, and (2) quenching chain-initiating catalysts in order to remove reactive oxygen or nitrogen species initiators [18].

This study determined the scavenging of stable radicals (i.e., DPPH and cation radical ABTS+), and the reducing potential of an antioxidant via the FRAP assay. The stable radical scavenging capacity of the antioxidants can be obtained by receiving an electron or hydrogen from the antioxidant or sprout samples. These radicals are stable and suitable models that are strongly absorbed in the visible region, and, thus, can be measured by spectrophotometry based on the absorption change. DPPH and ABTS+ scavenging assays are commonly used models because they are simple, rapid, sensitive, and consistently reproducible. The mechanism of antioxidant action in DPPH assay is through donating hydrogen to reduce the stable radical DPPH to the non-radical diphenyl-picrylhydrazine (DPPH-H) and ABTS+ radicals’ scavenging activity by single-electron transfer. The extract or compound contains molecular structures that bear active hydroxyl groups, such as polyphenols and flavonoids, which are potent radical scavengers [19]. The antioxidant activities of plant extracts were enhanced by the presence of phenolic compounds, flavonoids, alkaloids, anthocyanin compounds, and other secondary metabolites [20,21].

Secondary plant metabolites, produced in response to different stresses for the defensive mechanisms of plants, have been reported to possess many biological activities [22]. In our study, methanol was selected for the extraction of nine different sprouts. The polarity of the extraction solvent and the solubility of chemical constituents in the extraction solvent lead to different types of extraction compounds from plant materials. Based on many reports, methanol is one of the common solvents for extraction, producing a higher percentage yield of phenolic compounds than other solvents [23,24,25]. Examples of notable secondary metabolites extracted by polar solvent are as follows. Gallic acid, a phenolic compound containing three hydroxyl groups at positions three, four, and five, acts as an antioxidant, an antineoplastic agent, an apoptosis inducer, a gene protector, and an arachidonate 15-lipoxygenase inhibitor to protect human spermatozoa against oxidative stress [26,27]. Syringic acid is a dimethoxybenzene derivative of gallic acid. This substance was suggested to have a variety of biological effects, including anti-oxidant and anti-nitrosant capabilities, as well as anti-cancer, anti-bacterial, anti-inflammatory, and anti-diabetic activity [28,29]. Chlorogenic acid is a polyphenol found in coffee and black tea, which is an ester of caffeic acid and quinic acid. It exerts antioxidant and chemopreventive properties, preventing the development of cancer by scavenging free radicals, preventing DNA damage, and promoting the expression of genes related to immune system activation and increasing the activation and proliferation of cytotoxic T-lymphocytes, macrophages, and natural killer cells [30,31]. Caffeic acid, also known as a hydroxycinnamic acid with hydroxyl groups substituting for the phenyl ring at positions three and four, functions as an antioxidant, inhibiting histone deacetylase, arachidonate 15-lipoxygenase, glutathione transferase, and arachidonate 5-lipoxygenase [32,33]. Vanillin belongs to benzaldehydes that contain methoxy and hydroxy substituents at positions three and four, respectively. It acts as an anti-oxidant, anti-convulsant, anti-inflammatory, and flavoring [34,35]. Rutin is quercetin that has had the hydroxyl group at position C-3 replaced with sugar groups such as glucose and rhamnose and is known as an anti-oxidant [36,37].

Based on in vitro chemical screening of phytochemicals using a spectroscopic method, the total contents of phenolic, flavonoids, anthocyanin, chlorophyll, and alkaloids have been identified and quantified in our selected sprouts. They have been reported to exert various biological activities. Phenolics act as anti-oxidants by interacting with free radicals through different reactions. The hydroxyl group of phenolic compounds is responsible for anti-oxidant activity [38,39,40,41]. Both phenolic and flavonoid compounds are significant anti-oxidants due to their capacity to donate hydrogen atoms to free radicals and deactivate free radicals. They also possess the perfect structural qualities for free radical scavenging [42]. Anthocyanin and chlorophyll have also been reported to contribute to anti-oxidant activity in many plants [43,44]. Chlorophyll provides health advantages and helps prevent chronic diseases such as coronary heart disease, various cancers, and obesity [45,46,47]. Chlorophyll a and its breakdown products are widely used as anti-inflammatory agents and to prevent the uptake of carcinogens and carcinogenesis [48,49]. Alkaloid has been reported to exhibit anti-oxidant activity [50], anti-bacterial activity [51,52], anti-malarial activity [53], an anti-inflammatory effect [54,55], and anti-diabetic [56] and anti-cancer activities [57].

Some reports of biological activity and compounds have been published on the nine sprouts used in this study. Sunflower seeds and sprouts have significant anti-oxidant, anti-bacterial, anti-inflammatory, anti-hypertensive, wound-healing, and cardiovascular effects due to their phenolic compounds, flavonoids, polyunsaturated fatty acids, and vitamins [58]. Additionally, they comprised proline, chlorophyll, carbohydrates, proteins, and lipids. In ethnomedicine, they have been used to cure a variety of diseases, including whooping cough and bronchial, laryngeal, and pulmonary infections, as well as heart disease [59]. In the middle of flowering, the aerial parts of sunflowers are a rich source of phenolic compounds with anti-oxidant properties [60]. Mustard sprouts contain anti-oxidants that can neutralize reactive oxygen species without being transformed into destructive radicals [61]. The total phenolic compounds, total flavonoids, vitamins, and minerals are present in this plant [62]. The black sesame sprouts contained high levels of total phenolic and flavonoid content, proteins, unsaturated and saturated fatty acids, vitamins, minerals, and lignans such as sesamin, sesamol, sesamolin, and tocopherols [23]. Taiwanese morning glory sprouts exhibited anti-cancer, anti-bacterial, anti-oxidant, anti-calcification, and anti-mutagenic effects [63]. This plant has been used in Cambodia and Myanmar to cure febrile delirium and as a source of protein, vitamin, and mineral in chicken feed to promote growth [64]. The mung bean sprout has been reported to possess vitamin C, phenolics, carotenes, chlorophyll, and other nutrients [65]. The bioactivities of this sprout include detoxification, reducing the incidence of coronary heart disease, hypercholesterolemia, preventing hair and nail loss, and exhibiting antioxidant, anti-diabetic, and hypocholesterolemic properties [66]. Soybeans are an important crop for food security in Asia and also in Cambodia [67,68]. The phenolic, flavonoid, and anthocyanin profiles, as well as anti-oxidant activities, of soybean seeds were different across soybean varieties. Colored radish sprouts showed scavenging activities in the ABTS assay [69,70]; anti-microbial activity against *E*. *coli*, *S*. *pneumonia*, and *S*. *aureus*; and antiproliferation in the HT29 (colon cancer) and MCF7 (breast cancer) cell lines. The bioactive compounds in the colored radish sprouts determined by HPLC were anthocyanins such as pelargonidin, cyanidin, and delphinidin [71]. A previous study reported that flavonoids, phenolic acids, vitamins, and trace elements were abundant in radishes and were attributed to anti-oxidants. White radish sprouts were also reported for anti-oxidant activities via DPPH and FRAP assays. Asian white radishes possess anti-cancerous and anti-inflammatory properties in their edible solid taproot. Radish sprouts and mustard contain derivatives of hydroxycinnamic acids, including sinapic acid, chlorogenic acid, and flavonols [72]. Green pea pods were found to have a flavonoid and phenolic content, as well as other antioxidant activities according to the DPPH, FRAP, and ABTS assay [73,74].

HPLC analysis shows that the sprouts expressed various phytochemical compounds [75,76]. However, HPLC conditions and extraction methods can affect the presence and content of the extracted compounds [77,78]. Moreover, other unidentified peaks also appeared in our chromatogram. This study has some limitations; not all of the compounds in the extraction samples were identified by comparing them with the standard compounds or from the chemical screening of phytochemical analysis. Moreover, the lack of identical compounds between plant collections and the present study could be due to natural diversity and/or a difference in the growing conditions in the location of the collection. The presence of significant amounts and types of bioactive components in the sprouts under the present study and the quantity determined based on the selected solvent for the extraction process ensures its unequivocal recommendation for use in the pharmaceutical and nutraceutical sectors. However, more structured elucidation should be performed to disclose other compounds and a more detailed study of antioxidant activity to create a nutritional and medicinal reference for these sprouts and to evaluate their health benefits.

## 4. Materials and Methods

### 4.1. Material and Reagents

Analytical grade methanol was purchased from Merck KGaA (Darmstadt, Germany). HPLC grade methanol and acetonitrile were purchased from RCI Labscan V.S. CHEM HOUSE (Bangkok, Thailand). Syringic acid was purchased from Thermo Scientific (Fair Lawn, NJ, USA). Chlorogenic acid was purchased from AK Scientific (Union, CA, USA). Rutin was obtained from HiMedia Laboratories Pvt. Ltd. (Mumbai, India). Vanillin was purchased from Acros Organic, Janssen Pharmaceuticalaan 3a (Geel, Belgium). Caffeic acid was obtained from Thermo Scientific (Fair Lawn, NJ, USA). Gallic acid was purchased from Acros Organic, Janssen Pharmaceuticalaan 3a (Geel, Belgium). Trolox was obtained from Thermo Scientific (Fair Lawn, NJ, USA). Quercetin was obtained from HiMedia Laboratories Pvt. Ltd. (Mumbai, India). Furthermore, 2,4,6-Tri(2-pyridyl)-s-triazine (TPTZ) was purchased from Thermo Scientific (Fair Lawn, NJ, USA); 1,1-Diphenyl-2-picrylhydrazyl Free Radical (DPPH) was purchased from Tokyo Chemical Industry Co., Ltd. (Tokyo, Japan); 2,2′-Azino-bis (3-ethylbenzothiazoline-6-sulfonic acid) diammonium salt (ABTS) was Chem Cruz (Dallas, TX, USA). The HPLC standard atropine was purchased from HPC Standards GmbH (Cunnersdorf, Germany). Folin-Ciocalteu’s phenol reagent, aluminum chloride, Iron(III) chloride hexahydrate, potassium sulfate, ferric (II) sulfate hexahydrate, and hydrochloric acid were purchased from Merck KGaA (Darmstadt, Germany). The other reageants were purchased from standard commercial suppliers.

### 4.2. Plant Extraction

The extraction was performed following the previous report [79]. First, 1 kg of seeds was soaked in 2 L of water for 4 h, then evenly spread out on a wet towel for 8 h. Next, the seeds were prepared by soil germination in mesh greenhouses and sprayed with water twice daily at room temperature (25–30 °C). In total, 9 sprouts were harvested at the ages of 4 to 7 days. Finally, the aerial part of the sprouts was cut and collected for the experiment when they were mature, 5 cm long, and had 2 leaves (at 4–7 days). They were *Helianthus annuus* L. (sunflower, SF); *Brassica juncea* (L.) Czern. (mustard, MT); *Sesamum indicum* L. (black sesame, BS); *Ipomoea aquatica* Forssk. (Taiwanese morning glory, TG); *Vigna radiata* (L.) R. Wilczek (mung bean, MB); *Glycine max* (L.) Merr. (soybean, SB); *Raphanus sativus* L. (white radish, WR); *Raphanus sativus* L. (colored radish, CR); and *Pisum sativum* L. (green pea, GP). The sprouts were gently washed to remove dirt. The excess water was drained and the sprouts were dried in an oven (Biobase, Shandong, China) at 30 °C for 2 h then chopped into small fragments. The dried plant (20 g) was transferred to 1% concentrated hydrochloric acid in methanol and ultrasonicated at 30 °C for 30 min before filtering. The solvent was removed by a rotary evaporator (IKA RV10, Selangor, Malaysia) at room temperature to obtain the dried crude residue. The crude extract was stored in the refrigerator at 4 °C.

### 4.3. Identification of Phenolics and Flavonoids Using HPLC

The phenolic and flavonoid contents in the sprout extracts were determined by high-performance liquid chromatography (HPLC) using the modified method from a previous study [77]. HPLC chromatograms can be found in the Supplementary Materials (Figures S1–S10). Analyses were performed using an LC−2030C3D quaternary pump (Shimadzu, Kyoto, Japan) equipped with a diode array detector (DAD), according to the method of [80]. The extracts were dissolved in methanol (HPLC grade), filtered through a 0.45 µm membrane filter, and injected into the GIST C18 shim-pack column (4.6 × 250 mm, 5 µm) (KTA Technologies Corporation, Tokyo, Japan). The column temperature was 38 °C. The injection volume was 20 µL using an autosampler. The flow rate of the mobile phase was 0.8 mL/min. The mobile phase consisted of 1% acetic acid in purified water (A) and acetonitrile (B). The gradient elution was performed as follows: from 0 to 5 min, linear gradient from 5 to 9% of acetonitrile; from 5 to 15 min, 9 to 11% of acetonitrile; from 15 to 22 min, linear gradient from 11 to 15% of acetonitrile; from 22 to 30 min, linear gradient from 15 to 18% of acetonitrile; and from 30 to 38 min and a re-equilibration period of 10 min with 5% of acetonitrile used between individual runs. The detector wavelength was set at 280, 320, and 370 nm. The compounds in the sample were identified by comparing their retention time and UV spectral matching to standards. The phenolic and flavonoid standards were gallic acid, chlorogenic acid, vanillin, caffeic acid, syringic acid, and rutin.

### 4.4. Total Chlorophyll Content (TCC)

The TCC was performed following the previous report [81]. The stock solution of the crude extract in methanol was prepared with the final concentration of 2.5 mg/mL. The TCC was detected at wavelengths of 645 and 663 nm by microplate spectrophotometer (Thermo Scientific^TM^ Multiskan^TM^ FC, Boston, MA, USA). The TCC was calculated using Equation (1):

Total chlorophyll content (mg/g) = (20.2 × A645) + (8.02 × A663)/1000(1)

### 4.5. Total Flavonoid Contents (TFC)

The TFC was determined per previous studies [82,83]. The crude extracts were prepared by dissolving 100 mg of the extract in 1000 µL of methanol. Briefly, 100 µL of the extract was mixed with 50 µL of 2% aluminum chloride as a buffer. Quercetin was dissolved in methanol (10 mg/mL), then diluted at various concentrations (10, 20, 30, 40, 50, and 60 µg/mL). The experiments were carried out with five replications. The absorbance of the test solution was measured at 400–415 nm with a microplate spectrophotometer (Thermo Scientific^TM^ Multiskan^TM^ FC, Boston, MA, USA). The TFC was calculated from a standard curve of quercetin (y = 0.014x − 0.0014, R² = 0.9907). The results expressed the total flavonoid content as milligrams of quercetin equivalent (QE)/g of the crude extracts.

### 4.6. Total Phenolic Content (TPC) by Folin Ciocalteu’s Reagent

The TPC was determined using the Folin-Ciocalteu method [84,85]. The crude extracts were dissolved in methanol (final concentration of 10 mg/mL). Briefly, 15 µL of extract was mixed with 120 µL of prepared Folin-Ciocalteu’s reagents and placed at room temperature away from light for 5 min. Subsequently, 120 µL of sodium carbonate buffer (pH 7.5) was added to the mixture and kept for another 90 min under the same condition. The absorbance of the blue solution of molybdenum (V) in Folin-Ciocalteu’s reagents was measured at 725 nm with the microplate spectrophotometer (Thermo Scientific^TM^ Multiskan^TM^ FC, Boston, MA, USA). Gallic acid was used as a positive control. The gallic acid solution was dissolved in methanol (10 mg/mL) and prepared at different final concentrations (10, 20, 30, 40, and 50 µg/mL). The experiments were carried out in five replicates. The standard curve of gallic acid was created from the plot between the absorbance of the blue solutions of molybdenum (V) against the gallic acid concentrations. TPC was calculated from a standard gallic acid curve (y = 0.0166x + 0.1135, R^2^ = 0.9908). The results were expressed as milligrams of gallic acid equivalent (GAE) per gram of crude extract.

### 4.7. Total Alkaloids Content (TALC)

The TALC was performed according to previous studies [83,86]. The solution of crude extracts was prepared by dissolving 10 mg in 1000 µL of 2N HCL. Briefly, 1000 µL of the extract was mixed with 5 mL of bromocresol 0.2 mM and 5 mL of the citrate phosphate buffer (pH 4.7). The solution mixture was added by 5 mL of chloroform and shaken vigorously. The solution mixture was incubated at room temperature for 30 min. The chloroform layer appeared below the layer of the sample/standard solution, which was collected for analysis at 420 nm using the UV-spectrometry (GENESYSTM 10S UV-Visible Spectrophotometer, Thermo Fisher Scientific, Madison, WI, USA). Atropine, a positive control, was dissolved in 2N HCL at different final concentrations (0.01–0.1 mg/mL). The experiments were carried out in five replications. The standard curve of atropine was created from the plot between the absorbance and the blank solutions, which do not contain any sample mixtures. The TALC was calculated from a standard curve of atropine (y = 5.9942x + 0.0479, R^2^ = 0.9997). The results are expressed as milligrams of atropine equivalent (AE) per gram of crude extracts.

### 4.8. Total Anthocyanin Content (TAC)

The TAC was evaluated using the pH differential method, as described in previous reports [77,87]. Briefly, the two different pH solutions were prepared as follows: (1) potassium chloride buffer (pH 1.0 KCl buffer) and (2) pH 4.5 sodium acetate buffer (pH 4.5 CH3CO2Na buffer). The extract in methanol was diluted 10 times with the 2 buffers, shaken under dark conditions for 15 min, and then centrifuged at 419× *g* at room temperature before measuring the absorbance at 510 nm and 700 nm using a microplate reader (Thermo Scientific^TM^ Multiskan^TM^ FC, Boston, MA, USA). The blank determination (DI water) was also performed. The total anthocyanin of the extract was calculated following Equation (2) in terms of cyanidin-3-glucoside. The results were expressed as milligrams of equivalent cyanidin-3-glucoside per gram of crude extract and the average of the four replicates.

Total anthocyanin content (mg/g) = [Adiff × Mw × DF × 1000]/[ε](2)

- Adiff is (A510–A700) pH 1.0 − (A510–A700) pH 4.5;
- Mw is the molecular weight of cyanidin–3–glucoside (g/mol);
- DF is the dilution factor;
- ε is the molar extinction coefficient for 26,900 L mol^−1^ cm^−1^.

### 4.9. Antioxidant Capacity

#### 4.9.1. DPPH Radical Scavenging Activities

The DPPH assay was based on hydrogen atom transfer (HAT) reactions. In addition, 2,2-Diphenyl-1-picrylhydrazyl or DPPH will generate a stable free radical with an unpaired electron, delocalized throughout the molecule, producing stabilized molecules [84,88]. Briefly, various concentrations of the extracts dissolved in methanol (between 10 and 1000 g/mL) and the DPPH reagents were added and mixed in a 1:1 ratio in the 96-well plates for 30 min in the dark at room temperature. The loss of absorbance of the DPPH radical at 515 nm was measured using a microplate spectrophotometer (Thermo Scientific^TM^ Multiskan^TM^ FC, Boston, MA, USA). Trolox, a standard antioxidant, was used as a positive control. The linear curve (y = 0.0051x + 0.0225, R² = 0.9939) was obtained from the plot between the Trolox concentration and the DPPH radical scavenging power. The experiments were carried out in four replicates. The DPPH radical scavenging capacity was represented as the percentage of DPPH radical inhibition at 50% (IC_50_). The percentage of inhibition or % of the scavenging effect of DPPH was calculated following Equation (3):

%DPPH scavenging effect = [Abs of control − Abs of sample]/[Abs of control] × 100(3)

- Abs of control = absorbance of control or a reaction mixture in the absence of antioxidant of sample.
- Abs of sample = absorbance of the reaction mixture in the presence of sample.

#### 4.9.2. ABTS Radical Scavenging Activity

The 2,2′-azino-bis-3-ethylbenzthiazoline-6-sulphonic acid (ABTS) assay measures the antioxidant scavenging activity of ABTS free radicals generated by potassium persulfate in the aqueous phase [85,89]. ABTS and potassium persulfate were dissolved in distilled water with final concentrations of 7 mM and 2.45 mM, respectively. The ABTS radical cation produced by the addition of both solutions in a 1:0.5 ratio was then incubated at room temperature for 12 h in a dark room. The ABTS solution was diluted in ethanol to give an absorbance of 0.7 ± 0.02 at 743 nm. Various concentrations of sprout extract (50–1000 µg/mL) were added to 1 mL of diluted ABTS solution in a ratio of 1:100 and incubated for 30 min. The absorbance was measured at 734 nm using a microplate spectrophotometer (Thermo Scientific^TM^ Multiskan^TM^ FC, Boston, MA, USA). Trolox was used as a standard solution (0.1–0.8 mM). The result of the ABTS radical scavenging was expressed as an IC_50_ value (inhibition at 50%) or as a percentage of inhibition using Equation (4).

%ABTS scavenging effect = [Abs of control − Abs of sample]/[Abs of control] × 100(4)

- Abs of control = absorbance of control or a reaction mixture in the absence of antioxidant of sample.
- Abs of sample = absorbance of the reaction mixture in the presence of sample.

#### 4.9.3. Reducing Antioxidant Power based on FRAP Assay

The FRAP assay describes the ferric-reducing antioxidant power. This assay method measures the reduction in the ferric ion Fe^3+^-TPTZ complex to Fe^2+^-TPTZ [89,90]. Various concentrations of each extract, ferrous sulfate, and positive control such as quercetin were dissolved with methanol. The FRAP reagent comprised 300 mM of acetate buffer (pH 3.6), 10 mM of 2,4,6-tripyridyls-triazine (TPTZ) solution, and 20 mM of FeCl3.6H2O in a 10:1:1 ratio. The reaction mixture was pipetted into each well of a 96-well plate and incubated for 30 min in the dark at room temperature. The absorbance of the colored product (ferrous tripyridyltriazine complex) was measured at 593 nm with the microplate spectrophotometer (Thermo Scientific^TM^ Multiskan^TM^ FC, Boston, MA, USA) against a blank with methanol. The experiments were tested in five replicates. The standard curve of ferrous sulfate with various concentrations (30, 35, 40, 45, 100, 150, and 200 mM) was linear (y = 0.0013x + 0.0049, R^2^ = 0.9949). Quercetin was stated as a positive control with various final concentrations (10, 20, 30, and 40 µg/mL) and was linear (y = 0.0101x + 0.0601, R² = 0.9917). The FRAP value was calculated from the standard curve and expressed in mmol Fe^2+^ per gram of extract (mmol Fe^2+^/g extract).

### 4.10. Stastistical Analysis

The results are reported as the mean ± standard deviation (SD). Statistical analyses between samples were performed using nonparametric tests followed Kruskal-Wallis tests (SPSS version 26, SPSS Inc. Armonk, NY, USA). A significant difference was set at *p* < 0.05. Correlation analysis was performed using SPSS Version 26, SPSS Inc., Armonk, NY, USA. Principal component analysis (PCA) of bioactive components between sprouts and their antioxidant activities was performed using Python software version 3.10.5 (Python Software Foundation, Fredericksburg, VA, USA). The bioactive compounds were normalized before computing the PCA analysis. The PCA scores and loading variables were obtained from the PCA model. The PCA score plot was used to illustrate the correlation between bioactive compounds and its antioxidant. The loading plot indicated the correlation between the bioactive compounds and their antioxidant activity at 0.5 (moderate correlation) and 1.0 (strong correlation) as a black dashed circle and a red solid circle, respectively.

## 5. Conclusions

Based on the constitutive bioactive compounds and antioxidant activities of the nine species of sprouts studied, our findings suggest that the nine sprouts chosen for the study have health benefits beyond their nutritional value. Sunflower sprouts contained the highest total chlorophyll content. Soybean sprouts contained the highest total flavonoid content. The mustard sprouts were rich in total alkaloid content but contained the least total flavonoid content. DPPH and ABTS scavenging activity had a strong positive correlation to the total phenolic (TPC) and total anthocyanin (TAC) contents. The sprouts that exerted high TPCs and TACs also had high DPPH or ABTS scavenging activities (i.e., Taiwanese morning glory, colored radish, black sesame, white radish). Sunflowers, which were composed of the lowest TPC, had low DPPH and ABTS scavenging activities. The FRAP values had a strong positive correlation with vanillin and a moderate to weak correlation with the other compounds. The white radish that contained the highest vanillin, therefore, exhibited the highest FRAP value. The findings of our study could be viewed as exploratory and preliminary scientific evidence. Further research is needed to confirm the efficacy of safe sprout consumption in vitro, in vivo, and clinically, as well as to elucidate a more detailed structure and mechanism of action.

## Figures and Tables

**Table 1:** Detection wavelengths and retention times of standard phenolic and flavonoid compounds (*n* = 3).

Standard Compounds		Retention Time (min)		
		280 nm	320 nm	370 nm
Hydroxybenzoic Acids	Gallic acid (**1**)	7.24 ± 0.07	7.24 ± 0.07	Not detected
	Syringic acid (**4**)	23.47 ± 0.16	23.47 ± 0.16	Not detected
Benzaldehyde	Vanillin (**5**)	28.67 ± 0.04	28.67 ± 0.04	28.67 ± 0.04
Hydroxycinnamic Acids	Chlorogenic acid (**2**)	16.51 ± 0.28	16.51 ± 0.28	16.51 ± 0.28
	Caffeic acid (**3**)	21.57 ± 0.49	21.57 ± 0.49	21.57 ± 0.49
Flavonoids	Rutin (**6**)	36.15 ± 0.08	36.15 ± 0.08	36.15 ± 0.08

**Table 2:** Phenolic and flavonoid content in different sprouts detected by HPLC.

Compounds	Detected Amount (mg/g of Crude Extract)								
	SF	MT	BS	TG	MB	SB	WR	CR	GP
(i) Hydroxybenzoic acids									
Gallic acid (**1**)	ND	1.37 ± 0.01 ^d,B^	ND	ND	2.50 ± 0.02 ^c,A^	4.06 ± 0.01 ^a,A^	ND	ND	3.34 ± 0.01 ^b,B^
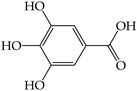									
Syringic acid (**4**)	ND	ND	0.464 ± 0.003 ^a,C^	0.052 ± 0.004 ^d,D^	0.089 ± 0.003 ^b,C^	0.019 ± 0.001 ^e,E^	0.069 ± 0.004 ^c,D^	0.066 ± 0.003 ^c,D^	0.070 ± 0.005 ^c,D^
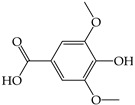									
(ii) Hydroxycinnamic acids									
Chlorogenic acid (**2**)	ND	2.39 ± 0.01 ^a,A^	ND	2.02 ± 0.01 ^e,A^	2.06 ± 0.00 ^d,B^	1.98 ± 0.01 ^f,B^	ND	2.21 ± 0.01 ^c,A^	2.38 ± 0.03 ^b,C^
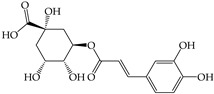									
Caffeic acid (**3**)	14.91 ± 0.08 ^a,A^	0.87 ± 0.01 ^c,D^	ND	0.60 ± 0.00 ^d,C^	ND	ND	0.35 ± 0.04 ^e,C^	ND	4.65 ± 0.07 ^b,A^
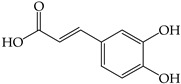									
(iii) Benzaldehydes									
Vanillin (**5**)	1.62 ± 0.01 ^c,B^	ND	0.94 ± 0.01 ^e,B^	1.6 ± 0.02 ^d,B^	ND	0.83 ± 0.01 ^f,D^	2.03 ± 0.01 ^a,A^	1.75 ± 0.01 ^b,B^	ND
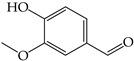									
(iv) Flavonoids									
Rutin (**6**)	ND	0.9 ± 0.00 ^d,C^	5.29 ± 0.02 ^a,A^	0.59 ± 0.01 ^f,C^	ND	1.40 ± 0.07 ^c,C^	0.79 ± 0.07 ^e,B^	0.54 ± 0.01 ^g,C^	2.49 ± 0.02 ^b,C^
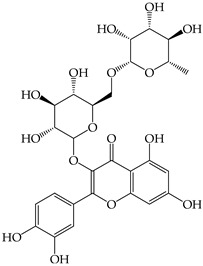									

ND = Not detected. Results expressed as the mean ± SD (*n* = 3). The compounds are numbered as they appear in the HPLC chromatograms. The different superscript letters between sprouts in the same row (wrote in small letters, a–g) or between the compounds at the same sprouts in the same column (wrote in upper case letters, A–E) indicates significantly different with *p* < 0.05.

**Table 3:** Antioxidant activities via DPPH (*n* = 4), ABTS scavenging activity, and FRAP assays (*n* = 5).

Samples	DPPH IC_50_ (µg/mL)	ABTS IC_50_ (µg/mL)	FRAP Value (mM FeSO_4_/g CE)
SF	1201.0 ± 38.47 ^g^	ND	0.023 ± 0.0020 ^d^
MT	1480.7 ± 154.93 ^h^	ND	0.024 ± 0.00005 ^d^
BS	486.3 ± 58.03 ^c,d^	527.4 ± 77.86 ^c^	0.032 ± 0.0034 ^c^
TG	283.6 ± 25.87 ^b^	108.2 ± 18.87 ^b^	0.031 ± 0.0004 ^c^
MB	556.5 ± 18.30 ^e^	ND	0.019 ± 0.0008 ^d^
SB	403.5 ± 36.78 ^c^	ND	0.0044± 0.0001 ^e^
WR	527.9 ± 14.87 ^d,e^	813.6 ± 57.10 ^d^	0.064 ± 0.0039 ^b^
CR	422.7 ± 43.05 ^c,d^	446.4 ± 45.75 ^c^	0.059 ± 0.0025 ^b^
GP	704.5 ± 23.80 ^f^	ND	0.031 ± 0.0012 ^c^
Trolox	32.6 ± 4.14 ^a^	44.0 ± 0.680 ^a^	-
Quercetin	-	-	0.56 ± 0.019 ^a^

Data are expressed as mean ± SD from five replicates. Different letters (a–h) indicates a significant different between samples (*p* < 0.05). ND = Not Detectable.

## Data Availability

The authors confirm that the data supporting the findings of this study are available within the article. The raw data were generated at Faculty of Pharmacy, University of Puthisastra and are available from V.S. upon reasonable request.

## Supplementary Materials

The following supporting information can be downloaded at: https://www.mdpi.com/article/10.3390/molecules28062874/s1, Figure S1: HPLC chromatograms of methanol; Figure S2: HPLC chromatograms of soybean sprout extract; Figure S3: HPLC chromatograms of Taiwanese morning glory sprout extract; Figure S4: HPLC chromatograms of green pea sprout extract; Figure S5: HPLC chromatograms of mung bean sprout extract; Figure S6: HPLC chromatograms of sunflower sprout extract; Figure S7: HPLC chromatograms of black sesame sprout extract; Figure S8: HPLC chromatograms of colored radish sprout extract; Figure S9: HPLC chromatograms of mustard sprout extract; Figure S10: HPLC chromatograms of white radish sprout extract.
