# Supplementary material for: Bioactive Compounds, Antioxidant Activities, and HPLC Analysis of Nine Edible Sprouts in Cambodia
Source: Molecules. 2023 Mar 22;28(6):2874. doi: 10.3390/molecules28062874 (PMC10059773; doi:10.3390/molecules28062874)
Supplement: Supplementary file 1 [file molecules-28-02874-s001.zip › molecules-2173938-supplementary.pdf]

Supplementary Materials

Article

Bioactive Compounds, Antioxidant Activities, and HPLC Analysis of Nine Edible Sprouts in Cambodia

Visessakseth So[1], Philip Poul[1], Sokunvary Oeung[2], Pich Srey[1], Kimchhay Mao[3], Huykhim Ung[3], Poliny Eng[3], Mengkhim Heim[4], Marnick Srun[5], Chantha Chheng[6], Sin Chea[7], Tarapong Srisongkram[8,9] and Natthida Weerapreeyakul [8,9,*]

[1] Division of Pharmacognosy, Faculty of Pharmacy, University of Puthisastra, Phnom Penh, 120204, Cambodia; svisessakseth@puthisastra.edu.kh (V.S.); pphilip.student@puthisastra.edu.kh (P.P.); spich2@puthisastra.edu.kh (P.S.)
[2] Division of Toxicology, Faculty of Pharmacy, University of Puthisastra, Phnom Penh, 120204, Cambodia; osokunvary1@puthisastra.edu.kh (S.O.)
[3] Division of Basic Pharmaceutical Sciences, Faculty of Pharmacy, University of Puthisastra, Phnom Penh, 120204, Cambodia; uhuykhim@puthisastra.edu.kh (H.U.); mkimchhay@puthisastra.edu.kh (K.M.); epoliny@puthisastra.edu.kh (P.E.)
[4] Division of Pharmacology, Faculty of Pharmacy, University of Puthisastra, Phnom Penh, 120204, Cambodia; hmengkhim@puthisastra.edu.kh (M.H.)
[5] Department of Technology Research and Development, National Institute of Science, Technology and Innovation, Phnom Penh, 120601, Cambodia; srun.marnick@misti.gov.kh (M.S.)
[6] Division of Pharmaceutical Technology, Faculty of Pharmacy, University of Puthisastra, Phnom Penh, 120204, Cambodia; cchantha@puthisastra.edu.kh (C.C.)
[7] Faculty of Pharmacy, University of Puthisastra, Phnom Penh, 120204, Cambodia; csin1@puthisastra.edu.kh (S.C.)
[8] Division of Pharmaceutical Chemistry, Faculty of Pharmaceutical Sciences, Khon Kaen University, Khon Kaen, 40002, Thailand; natthida@kku.ac.th (N.W.), tarasri@kku.ac.th (T.S.)
[9] Human High Performance and Health Promotion Research Institute, Khon Kaen University, Khon Kaen, 40002, Thailand
* Correspondence: natthida@kku.ac.th; Tel.: +66 43 202 378

Supplementary Material, Figure S1

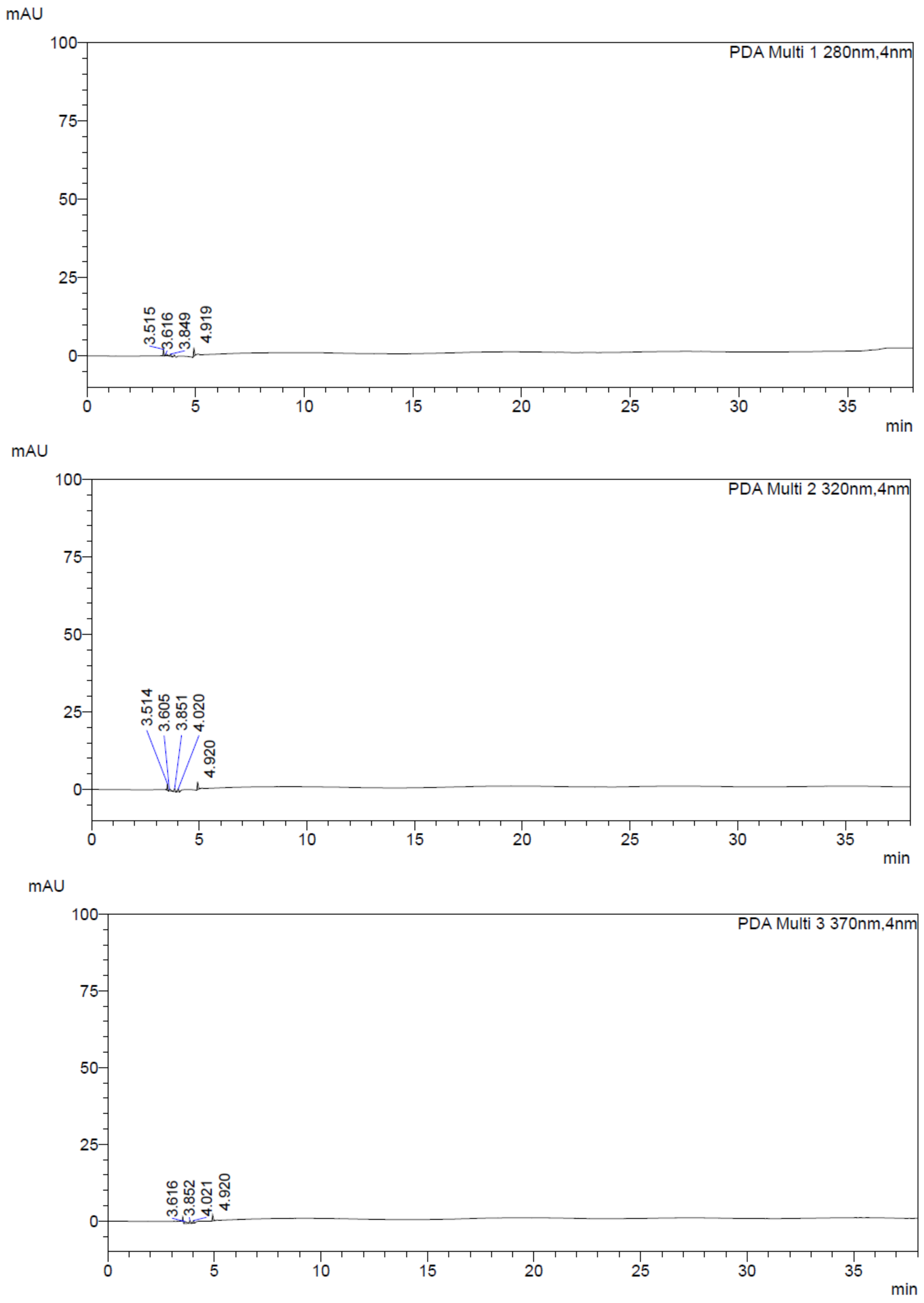

Figure S1. HPLC chromatograms of methanol were detected at 280, 320, and 370 nm.

Supplementary Material, Figure S2

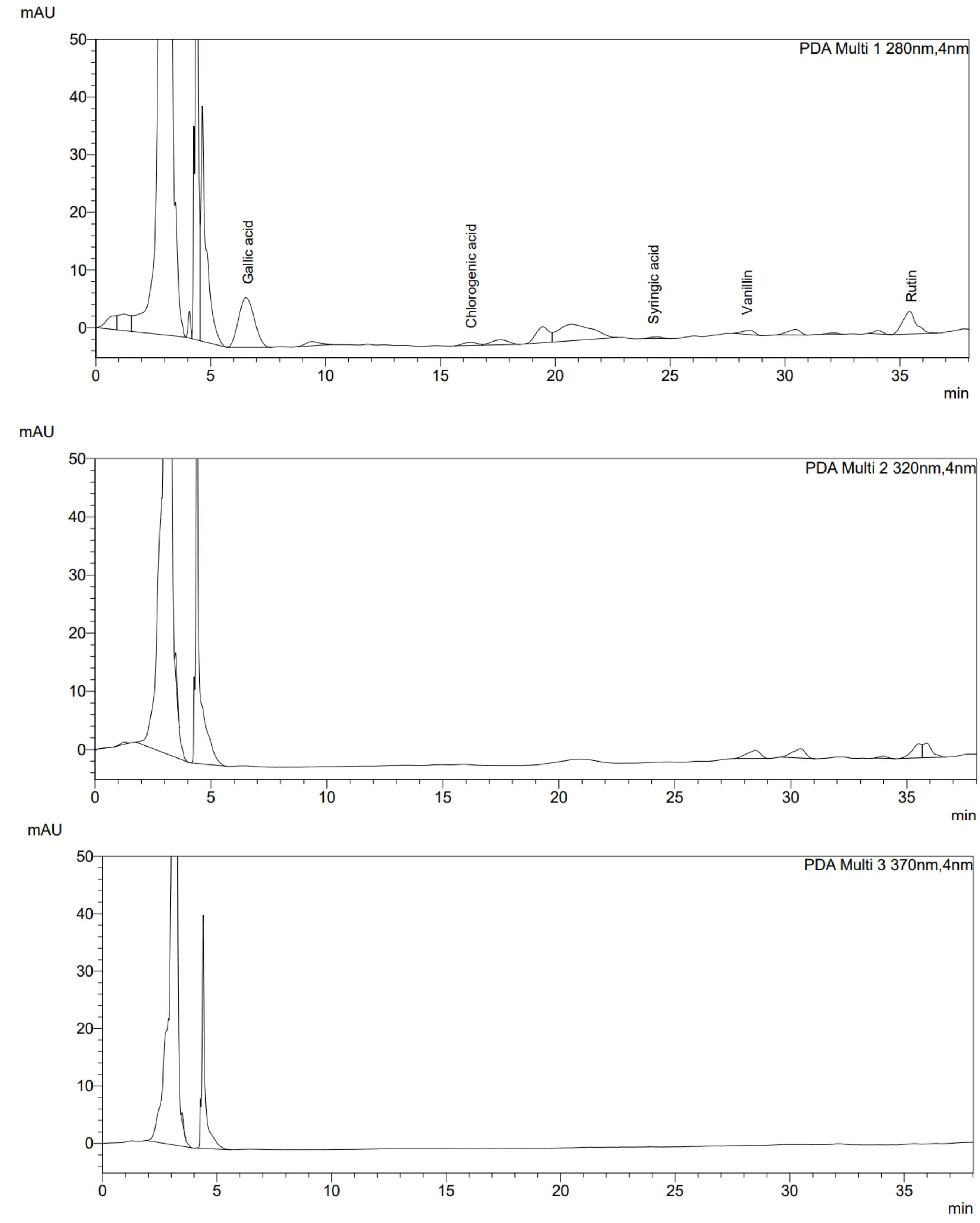

Figure S2. HPLC chromatograms of soybean sprout extract at 10 mg/ml in methanol were detected at 280, 320, and 370 nm showing peaks for gallic acid, chlorogenic acid, syringic acid, vanillin, rutin, and other unknown peaks.

Supplementary Material, Figure S3

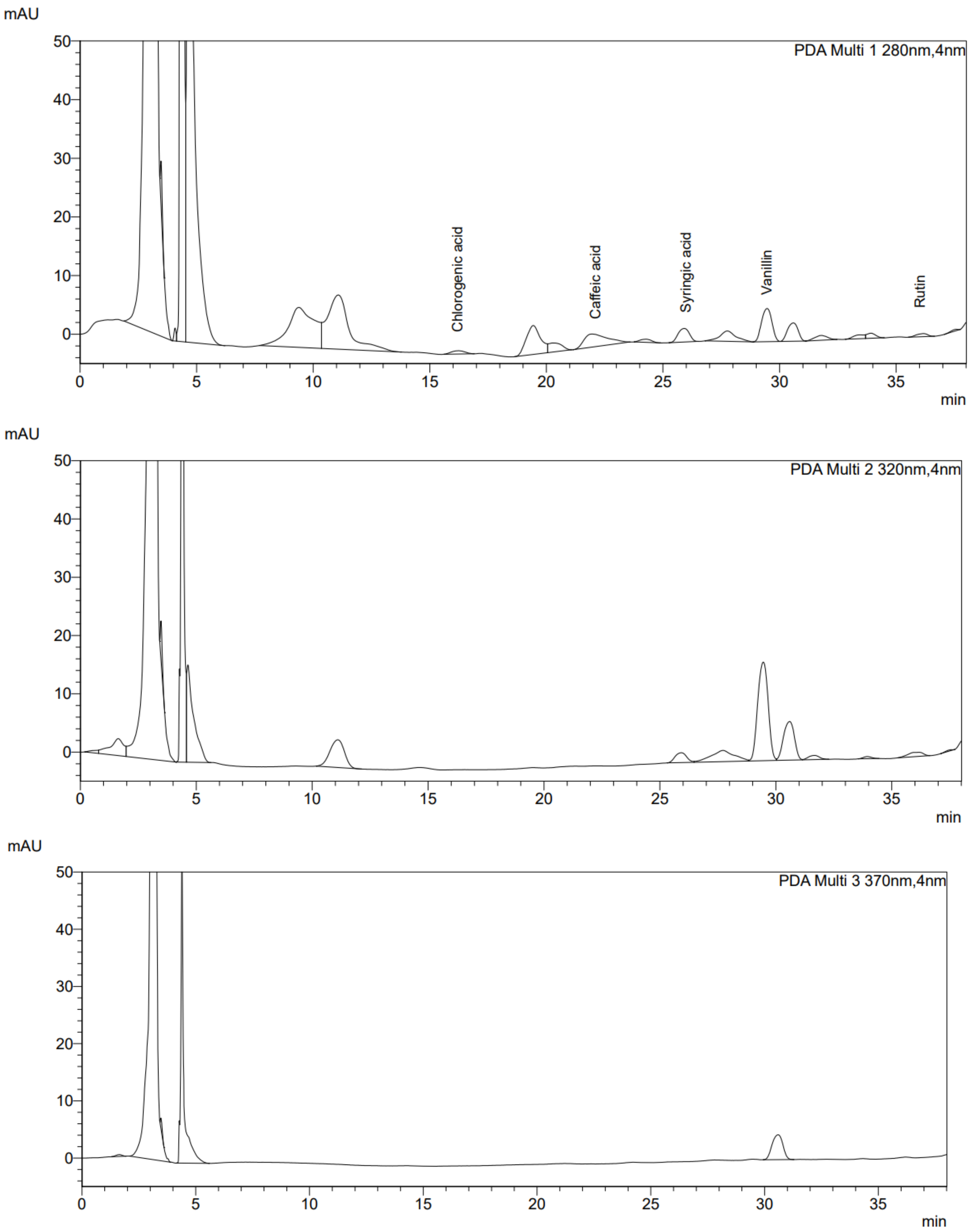

Figure S3. HPLC chromatograms of Taiwanese morning glory sprout extract at 10 mg/ml in methanol were detected at 280, 320, and 370 nm showing peaks for chlorogenic acid, caffeic acid, syringic acid, vanillin, rutin, and other unknown peaks.

Supplementary Material, Figure S4

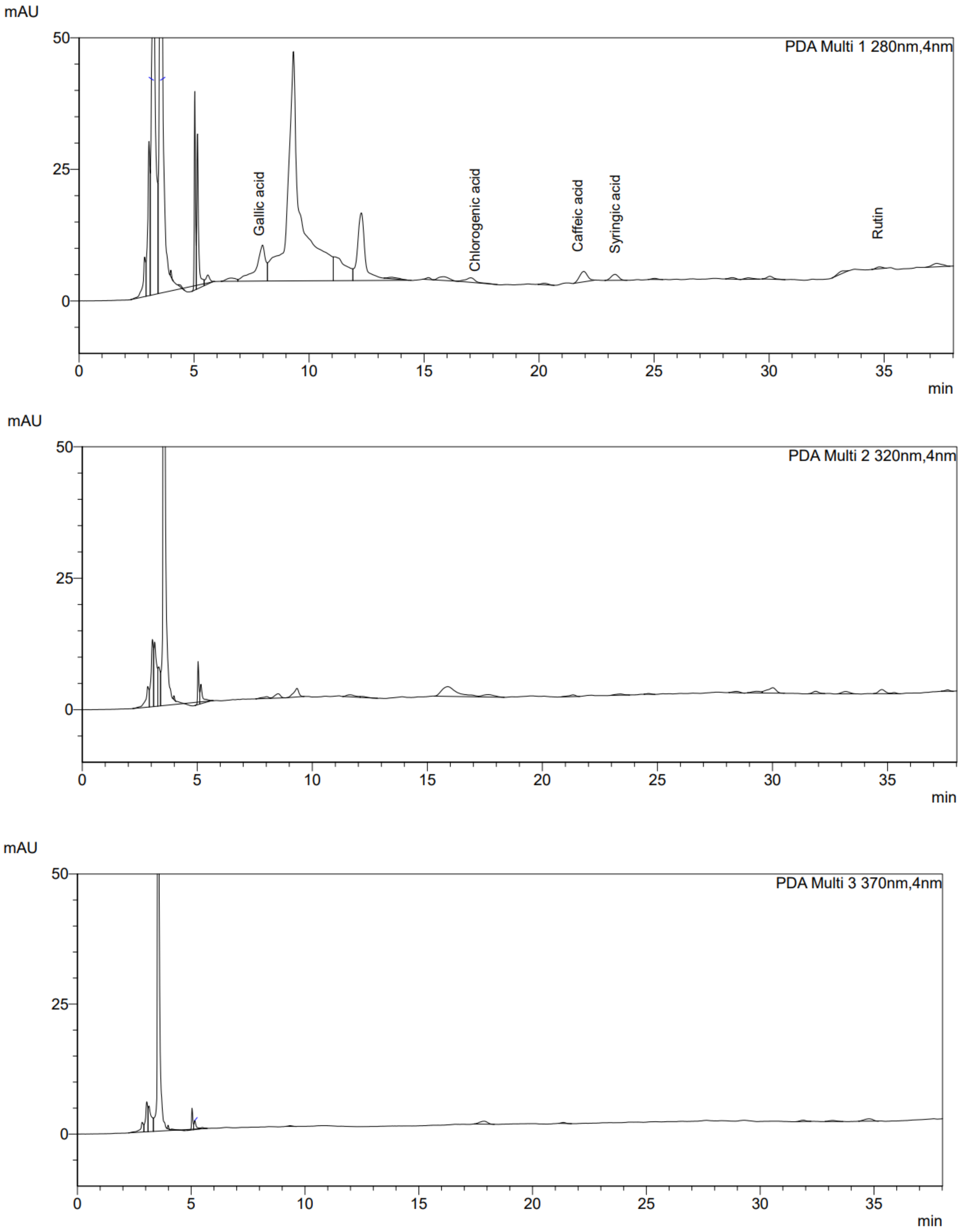

Figure S4. HPLC chromatograms of green pea sprout extract at 10 mg/ml in methanol were detected at 280, 320, and 370 nm showing peaks for gallic acid, chlorogenic acid, caffeic acid, syringic acid, rutin, and other unknown peaks.

Supplementary Material, Figure S5

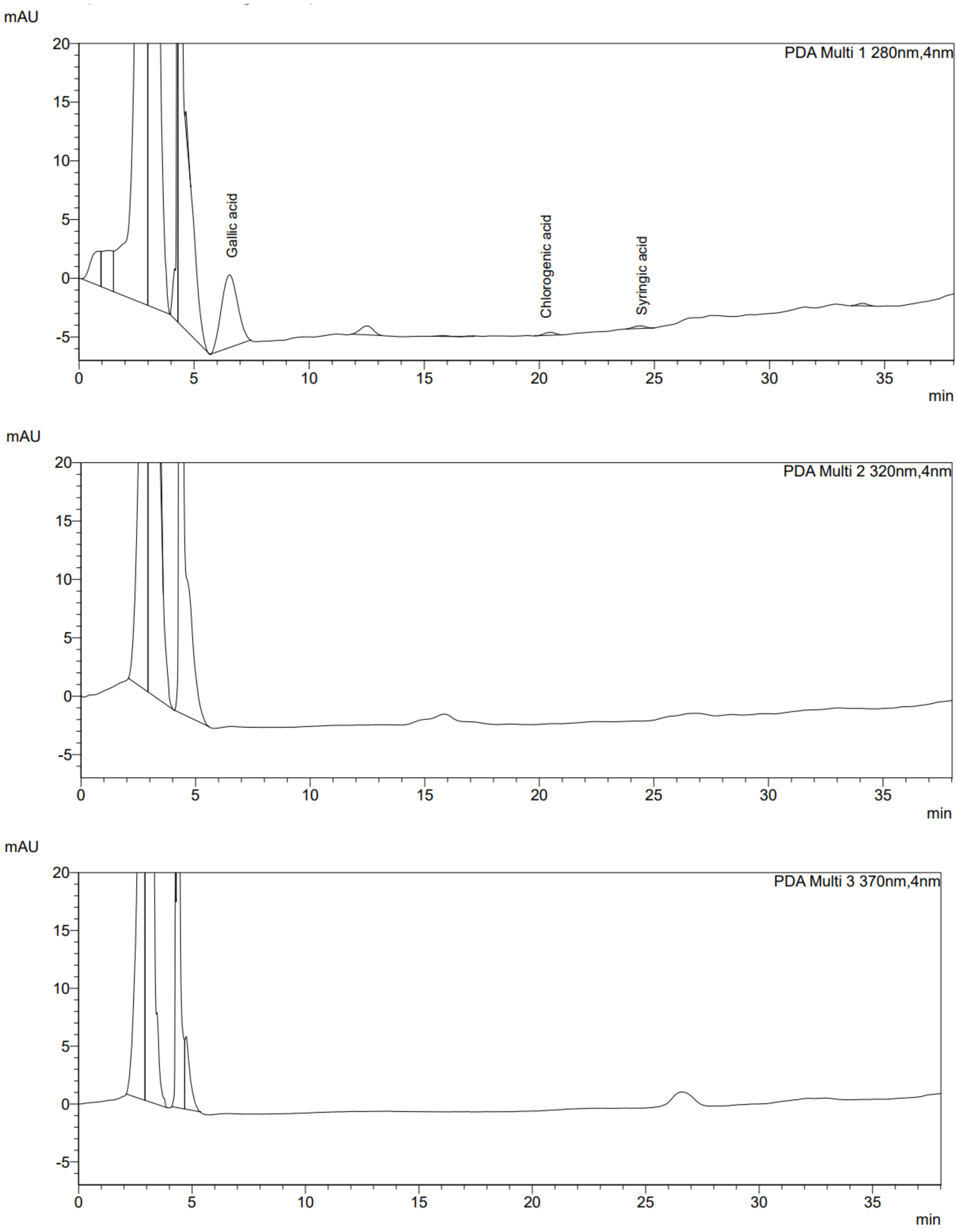

Figure S5. HPLC chromatograms of mung bean sprout extract at 10 mg/ml in methanol were detected at 280, 320, and 370 nm showing peaks for gallic acid, chlorogenic acid, syringic acid, and other unknown peaks.

Supplementary Material, Figure S6

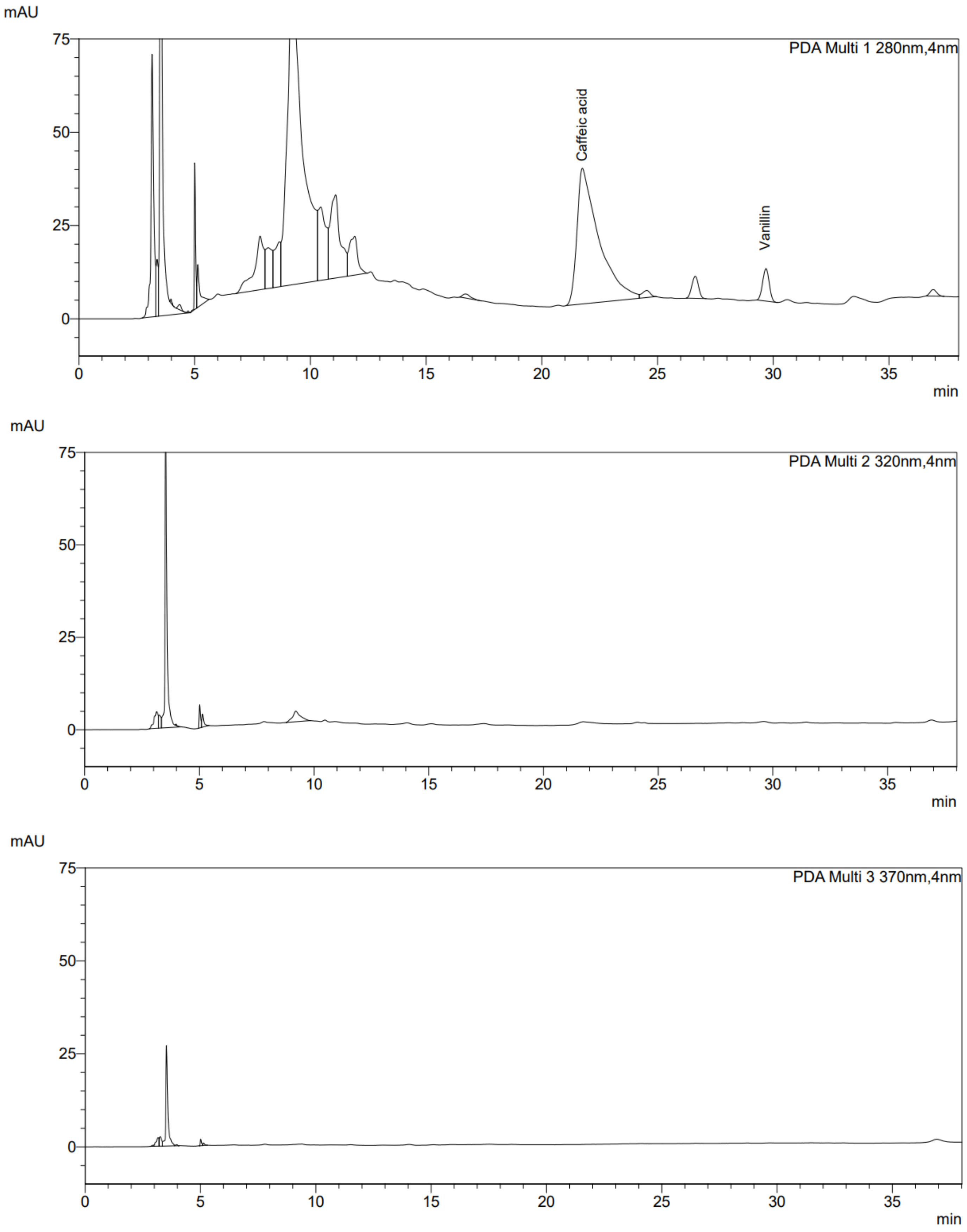

Figure S6. HPLC chromatograms of sunflower sprout extract at 10 mg/ml in methanol were detected at 280, 320, and 370 nm showing peaks for caffeic acid, vanillin, and other unknown peaks.

Supplementary Material, Figure S7

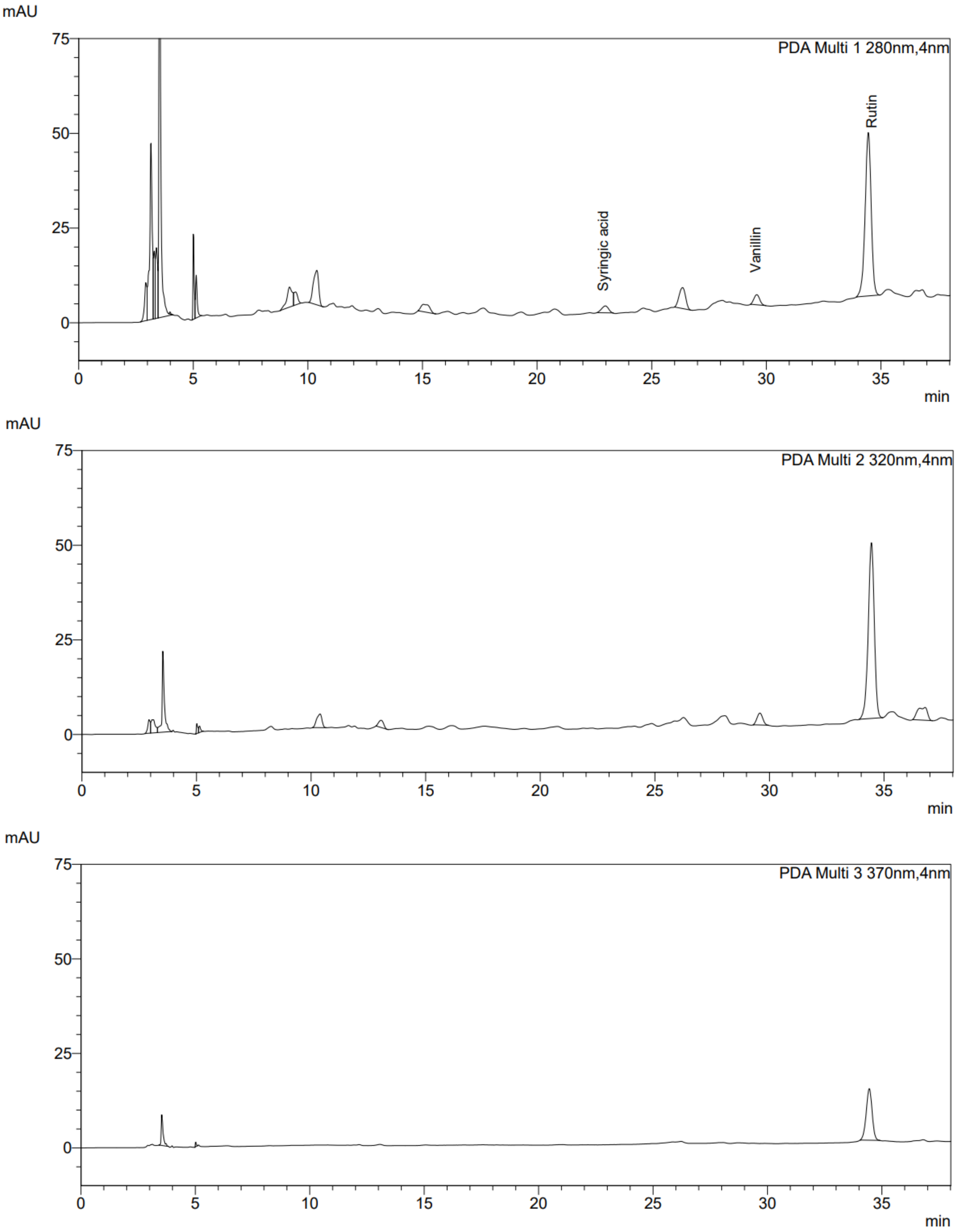

Figure S7. HPLC chromatograms of black sesame sprout extract at 10 mg/ml in methanol were detected at 280, 320, and 370 nm showing peaks for syringic acid, vanillin, rutin, and other unknown peaks.

Supplementary Material, Figure S8

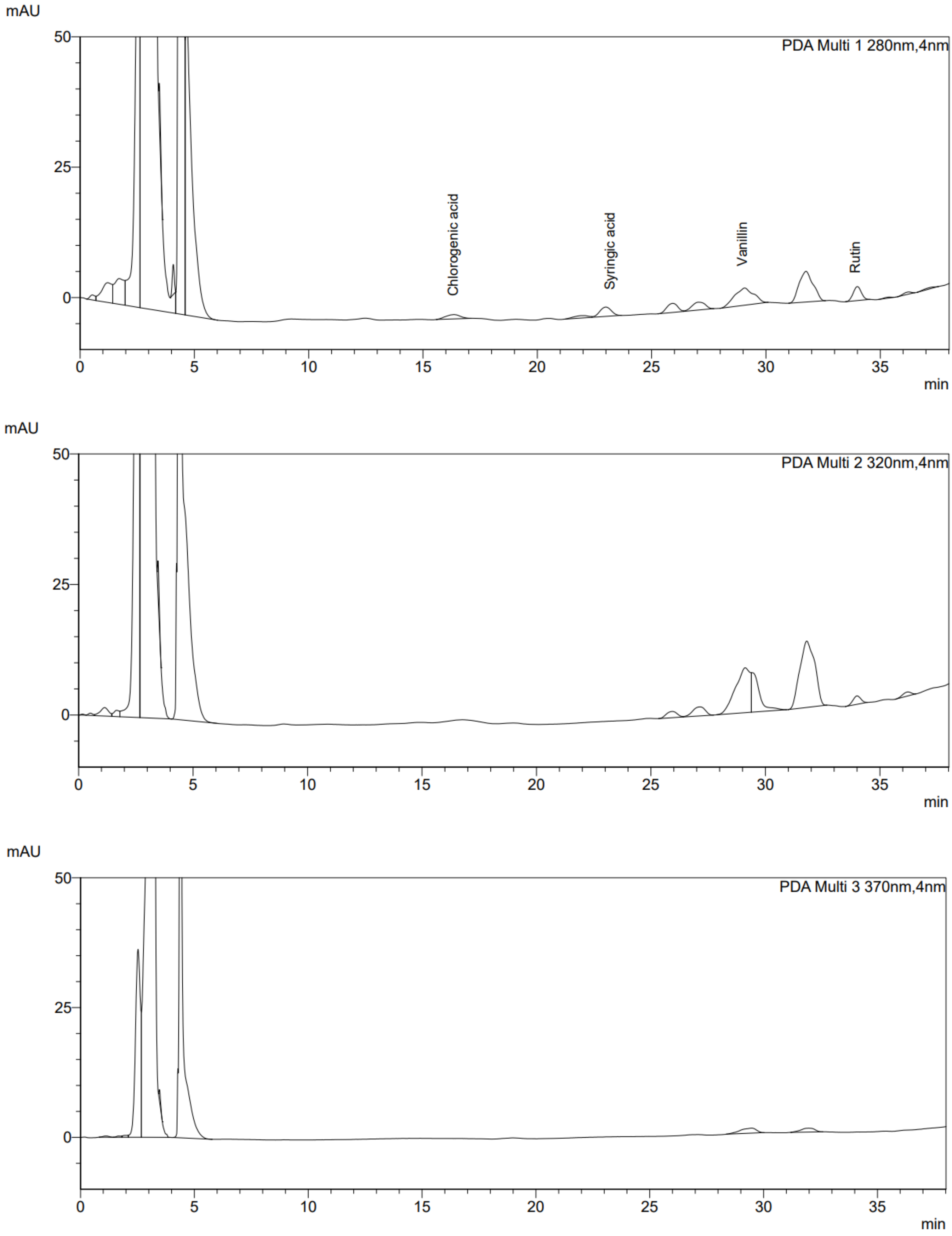

Figure S8. HPLC chromatograms of colored radish sprout extract at 10 mg/ml in methanol were detected at 280, 320, and 370 nm showing peaks for chlorogenic acid, syringic acid, vanillin, rutin, and other unknown peaks.

Supplementary Material, Figure S9

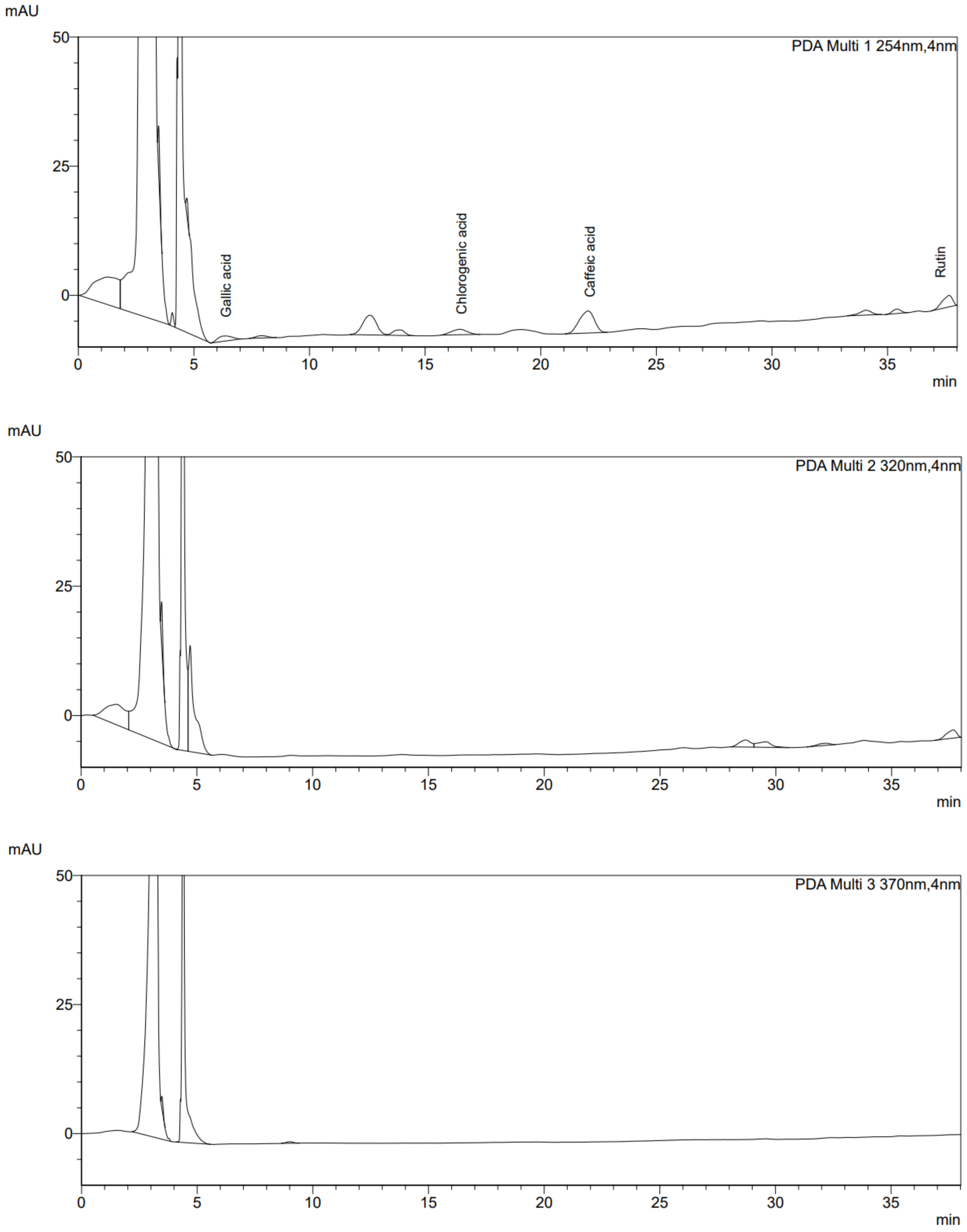

Figure S9. HPLC chromatograms of mustard sprout extract at 10 mg/ml in methanol were detected at 280, 320, and 370 nm showing peaks for gallic acid, chlorogenic acid, caffeic acid, rutin, and other unknown peaks.

Supplementary Material, Figure S10

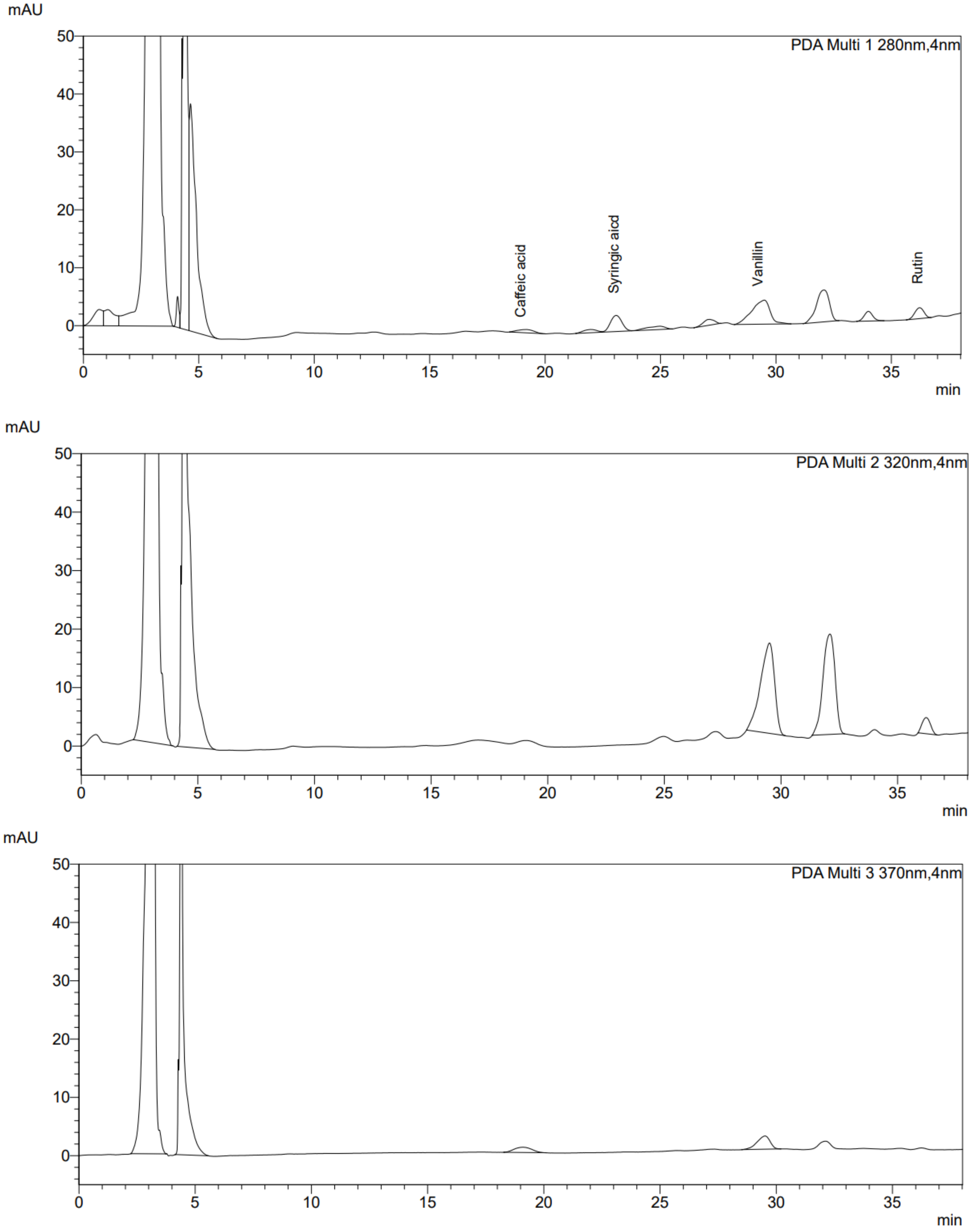

Figure S10. HPLC chromatograms of white radish sprout extract at 10 mg/ml in methanol were detected at 280, 320, and 370 nm showing peaks for syringic acid, caffeic acid, vanillin, rutin, and other unknown peaks.
